# Synthesis-Sequence-Controlled Surface and Electronic Structure in Pd–Ag–Ni(OH)_2_/C Electrocatalysts for Efficient Formic Acid Oxidation

**DOI:** 10.1021/acsomega.5c12585

**Published:** 2026-05-12

**Authors:** Maria E. S. C. Argôlo, Caio V. S. Almeida, Connor Sherwin, Andrea E. Russell, Katlin I. B. Eguiluz, Giancarlo R. Salazar-Banda

**Affiliations:** † Laboratory of Electrochemistry and Nanotechnology, Institute of Technology and Research, 49.032-490 Aracaju, Sergipe, Brazil; ‡ Chemical Engineering Department, 67896 Tiradentes University, 49.032-490 Aracaju, Sergipe, Brazil; § Process Engineering Postgraduate Program, 67896 Tiradentes University, 49.032-490 Aracaju, Sergipe, Brazil; ∥ School of Chemistry, 7423 University of Southampton, University Road, Southampton SO17 1BJ, United Kingdom

## Abstract

The development of efficient and durable electrocatalysts for the formic acid oxidation reaction (FAOR) is central to the progress of direct formic acid fuel cells (DFAFCs). Here, we investigate how the synthesis sequence and reduction pathway influence the surface and electronic structure of PdAgNi­(OH)_2_/C nanocomposites and, consequently, their FAOR performance in acidic media. Binary Pd/Ni­(OH)_2_ catalysts with Pd/Ni­(OH)_2_ mass ratios of 30:70, 50:50, and 70:30 were first screened, revealing 50:50 as the optimal composition. Partial substitution of Pd by Ag (Pd_40_Ag_10_ and Pd_30_Ag_20_ on Ni­(OH)_2(50)_/C) was then combined with either sequential or simultaneous NaBH_4_-assisted reduction. Structural characterization by XRD, TEM, and XANES/EXAFS show that simultaneous coreduction tightens the Pd–Ag–Ni interfacial coupling, enhances Pd dispersion, and increases the contribution of Pd–O and Pd–Ni scattering paths, indicative of strong metal–oxide interactions. Electrochemical measurements demonstrate that the Pd_30_Ag_20_Ni­(OH)_2(50)_/C catalyst prepared by simultaneous reduction exhibits the highest mass activity toward FAOR (6164 mA mg_Pd_^–1^), a ca. 23-fold enhancement over commercial Pd/C, together with improved stability under potential cycling. These results demonstrate that controlling the synthesis sequence is an effective method for tuning the interfacial electronic structure of multicomponent Pd-based catalysts, providing practical guidelines for designing FAOR electrocatalysts for DFAFCs and related liquid-fuel energy conversion devices.

## Graphical abstract

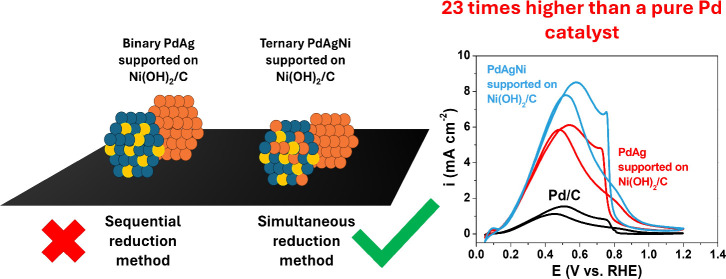

## 1 Introduction

The quest for alternative and sustainable energy sources is crucial in today’s world. Fuel cell technology, propelled by scientific and technological advancements, has become a significant modern energy source.^1^ Fuel cells are considered sustainable energy conversion systems and play a pivotal role in advancing the hydrogen economy.^2,3^

Formic acid has emerged as a liquid fuel candidate for polymer electrolyte membrane fuel cells (PEMFCs), serving as an alternative to hydrogen and methanol. As a stable, pumpable feed, it can provide a conductive medium at the anode, aiding proton transport within the electrode environment.^2^ Formic acid offers logistical advantages over hydrogen in terms of transportation and storage. Moreover, it exhibits a lower rate of membrane crossover compared to methanol, potentially yielding better performance under equivalent conditions.^2^

Pd is widely used as an anodic catalyst for the formic acid oxidation reaction (FAOR). However, the catalytic efficiency of Pd is often compromised by the rapid poisoning of its surface due to the adsorption of intermediate species, such as CO, from the oxidation process. This degradation in activity is further intensified by the challenges associated with synthesizing well-dispersed, finely sized Pd catalysts. Furthermore, the high cost of Pd poses significant challenges to the commercial viability of direct formic acid fuel cells on a large scale.^4,5^

Integrating multiple components into catalysts has emerged as a prevalent approach to enhance their catalytic performance and durability. Introducing additional metals such as Ag, Au, Ni, Sn, and Cu modifies the electronic configuration of palladium (Pd), thereby reducing the adsorption strength of CO on Pd active sites.^6–11^ Specifically, Pd–Ag alloys have demonstrated superior electrocatalytic properties and increased resistance to deactivation, attributed to the strong synergistic effects between Pd and Ag.^6,12–15^ Computational studies using the Hammer–No̷rskov model reveal that the d-band center of Pd, which has a lattice parameter of 3.89 Å, shifts upward upon forming an alloy with Ag, which has a lattice parameter of 4.09 Å.^7^ This shift in the d-band center diminishes the binding affinity between Pd and adsorbed intermediates, thereby facilitating their removal. Additionally, the presence of Ag enhances the oxidation of these intermediates, further preventing the deactivation of Pd sites.^6^

Recently, Karatok and co-workers^13^ studied the decomposition pathways of formic acid on Pd–Ag alloys with different atomic configurations. They generated various Pd_*x*_Ag_1–*x*_ surface alloys on a Pd(111) single crystal. Using temperature-programmed reaction spectroscopy (TPRS) and density functional theory (DFT), they found that modified Ag domains create a new reaction pathway that selectively dehydrogenates formic acid. In contrast, Pd monomers surrounded by Ag exhibit reactivity similar to pristine Pd(111), producing CO and H_2_O, but bind CO less strongly, demonstrating enhanced resistance to CO poisoning. This research highlights that surface Ag domains, influenced by subsurface Pd, are crucial for the selective decomposition of formic acid, while surface Pd adversely affects selectivity.

Another strategy to enhance the catalytic properties of Pd-based catalysts focuses on the addition of metallic oxides or hydroxides, such as Ni­(OH)_2_, SnO_2_, and NiO. These additives facilitate the formation of active hydroxyl species (OH_ads_) on the catalyst surface, aiding in the oxidation of intermediates.^5,16–18^ Morphological changes in Ni­(OH)_2_ strongly affect catalytic performance. In particular, Ni­(OH)_2_ nanosheets tend to exhibit defect-rich structures that facilitate water dissociation upon adsorption. Furthermore, the nanosheets’ morphology can enhance their interaction with Pd nanoparticles, improving electronic effects.

Yang et al.^17^ reported a notable increase in catalytic activity when using amorphous Ni­(OH)_2_ to modify Pd for formic acid and EOR. They observed that the current densities achieved were significantly higher than those from commercial Pd/C catalysts. This enhancement is ascribed to Pd electronic tuning together with the ability of amorphous Ni­(OH)_2_ to supply surface hydroxide species that assist in removing adsorbed CO. In addition, synthesis conditions can tailor the physicochemical profile of Pd/Ni­(OH)_2_, most notably the dispersion of Pd on the Ni­(OH)_2_ phase. Almeida et al.^5^ prepared Pd/Ni­(OH)_2_/C with Pd/Ni­(OH)_2_ ratios of 30:70, 50:50, and 70:30 and evaluated them for ethanol oxidation in alkaline media. The best performance was obtained with 30% Ni­(OH)_2_ (i.e., 70:30), which maximized Pd exposure on the carbon support near Ni­(OH)_2_ nanosheets.

The reduction sequence of metallic precursors significantly affects the catalytic activity of multicomponent materials by determining the nanoparticles’ final structure, surface composition, and electronic properties. These structural variations directly influence the number and types of active sites available, the extent of alloying, and the resistance to deactivation. As a result, the catalyst’s performance can be finely tuned for specific reactions.^19,20^

The sequence in which metals are combined influences whether the resulting nanoparticles form an alloy, a core–shell structure, or a nonuniform distribution of metals. Each of these structures exposes different metal facets and surface compositions, which act as active sites for reactions. In a sequential reduction method, one metal is reduced first to create initial seeds or templates, onto which the second metal is later deposited. This approach allows for better control over the formation of core–shell architecture. In contrast, when both precursors are reduced simultaneously, the result is often a more homogeneous alloy structure.^21,22^

The different reduction kinetics associated with each precursor and the reduction order affect the final size and dispersion of the metal nanoparticles on the support material. Improved dispersion usually results in a larger active surface area, thereby enhancing catalytic activity.^19^

In this context, a systematic understanding of how the synthesis sequence and reduction pathway govern the structure and performance of multicomponent Pd–Ag–Ni­(OH)_2_/C catalysts for FAOR is still lacking. Here, we investigate the effect of Pd/Ni­(OH)_2_ mass ratio, partial substitution of Pd by Ag, and sequential versus simultaneous NaBH_4_-assisted reduction on the composition, surface structure, and FAOR activity of Pd–Ag–Ni­(OH)_2_/C nanocomposites. Binary Pd/Ni­(OH)_2_/C catalysts are first optimized and then used as platforms to introduce Ag. XRD, TEM, and XANES/EXAFS analyses are combined with electrochemical testing to correlate synthesis parameters with activity, mass utilization, and durability. By identifying the synthesis conditions that maximize the beneficial metal–oxide interactions, we establish synthesis-sequence engineering as a powerful lever to design Pd-based electrocatalysts for the formic acid oxidation reaction and related liquid-fuel oxidation processes.

## 2 Experimental Section

### 2.1 Materials

In this study, various chemical reagents sourced from Sigma-Aldrich Brazil were used without further purification. These included palladium­(II) chloride (PdCl_2_, 99% purity), nickel­(II) chloride hexahydrate (NiCl_2_·6H_2_O, 99% purity), silver nitrate (AgNO_3_), sodium chloride (NaCl, 99% purity), sodium borohydride (NaBH_4_, 98% purity), sulfuric acid (H_2_SO_4_, 98% purity), ascorbic acid (C_6_H_8_O_6_, 99% purity), potassium hydroxide (KOH, 90% purity), formic acid (99% purity), 2-propanol (99.5% purity), Nafion 117 solution, and carbon powder. The Vulcan XC-72 carbon powder was specifically procured from Cabot. All solutions were prepared with ultrapure water, exhibiting a resistivity of 18.0 MΩ cm at room temperature, using a Gehaka MS 2000 system for purification.

### 2.2 Synthesis of Binary Pd_*x*_/Ni­(OH)_2(1–*x*)_

The Pd/Ni­(OH)_2_/C catalysts were synthesized using mass ratios of 70:30, 50:50, and 30:70 between Pd and Ni­(OH)_2_, maintaining a total metal content of 20% by weight across all samples. For the synthesis of Ni­(OH)_2_ supported on carbon at concentrations of 30, 50, and 70% (denoted as Ni­(OH)_2(30)_/C, Ni­(OH)_2(50)_/C, and Ni­(OH)_2(70)_/C), the process began with dissolving appropriate amounts of NiCl_2_ in 50 mL of ultrapure water, followed by 30 min of stirring. The pH was adjusted to 12 by the dropwise addition of a 1.0 mol L^–1^ KOH solution to promote the formation of Ni­(OH)_2_.^1^ The solution was left to stand for 1 h. Subsequently, carbon powder (80 mg) was added, and the suspension was stirred for an additional 24 h to promote uniform deposition of Ni­(OH)_2_ onto the carbon. The resulting solid was collected by filtration, washed with ultrapure water, and dried at 70 °C for 4 h.

To obtain the Pd_*x*_/Ni­(OH)_2(*y*)_/C catalysts, Ni­(OH)_2_/C was suspended in 50 mL ultrapure water, after which a 0.22 mol L^–1^ Na_2_PdCl_4_ solution was introduced. The latter was prepared by dissolving 0.10 g PdCl_2_ and 0.066 g NaCl in 25 mL ultrapure water with continuous stirring for 12 h. Then, the suspension was sonicated for 30 min, heated to 80 °C, and subsequently, 50 mg of ascorbic acid was added while maintaining stirring for 24 h. The final product was then collected using the same filtration and drying process as described previously.

### 2.3 Synthesis of Ternary Pd_*x*_Ag_1–*x*_/Ni­(OH)_2(50)_ Catalysts by Sequential Reduction

The synthesis of Pd_40_Ag_10_/Ni­(OH)_2(50)_/C and Pd_30_Ag_20_/Ni­(OH)_2(50)_/C catalysts was carried out following a methodology similar to that described previously. Initially, 0.08 g of Ni­(OH)_2(50)_/C was dispersed in 50 mL of water. This was accompanied by the addition of varying volumes of Na_2_PdCl_4_ solution (0.22 mol L^–1^), specifically 2.6 mL for Pd_40_ and 2.0 mL for Pd_30_ configurations, and varying amounts of AgNO_3_, with 3.15 mg for Ag_10_ and 6.30 mg for Ag_20_. The mixture was subjected to ultrasonication for 30 min.

Subsequently, the suspension was heated to 80 °C, and 40 mg of NaBH_4_ was added under continuous stirring. The mixture was maintained at 80 °C for 3 h and subsequently stirred at room temperature for an additional 24 h. The resulting catalyst powder was recovered by vacuum filtration, thoroughly washed with ultrapure water and ethanol, and dried under ambient conditions. Across all catalyst formulations, the total metal loading was consistently maintained at 20 wt %.

### 2.4 Synthesis of Ternary Pd_30_Ag_20_Ni­(OH)_2(50)_ Catalysts by Simultaneous Reduction

The synthesis of Pd_30_Ag_20_Ni­(OH)_2(50)_/C catalysts was carried out following a methodology analogous to that described in Section 2.3, with the sole modification being the simultaneous addition of the Ni precursor (NiCl_2_) alongside the Pd and Ag precursors. Catalysts prepared via sequential reduction were denoted as Pd_*x*_Ag_1–*x*_/Ni­(OH)_2(50)_/C, whereas the catalyst synthesized via the simultaneous reduction approach is expressed as Pd_30_Ag_20_Ni­(OH)_2(50)_/C. This nomenclature is consistently applied throughout the manuscript. For full procedures and additional results of the physical and electrochemical characterization, see Supporting Information.

## 3 Results and Discussion

### 3.1 Physical Characterization

Figure 1 displays the XRD patterns for the Pd/C, Pd_50_/Ni­(OH)_2(50)_/C, Pd_50_Ni­(OH)_2(50)_/C, and Pd_*x*_Ag_1–*x*_/Ni­(OH)_2(50)_/C catalysts. All diffractograms exhibit a broad peak centered at approximately 2θ = 25°, which is attributed to the (002) plane of graphitic carbon, in accordance with PDF card no. 01-0646. Figure 1a shows that for Pd/C, five peaks located at 2θ = 39.9°, 46.2°, 67.9°, 81.8°, and 86.5° correspond to the (111), (200), (220), (311), and (222) planes (PDF no.: 01-087-0643), respectively, indicating the typical polycrystalline face-centered cubic (fcc) structure of Pd.^1,2^ Figures 1a and S1a also display diffraction peaks associated with the (100), (101), (102), (110), and (111) planes of the Ni­(OH)_2_ phase (PDF no.: 00-001-1047).^1,4,5^

**Figure 1:**
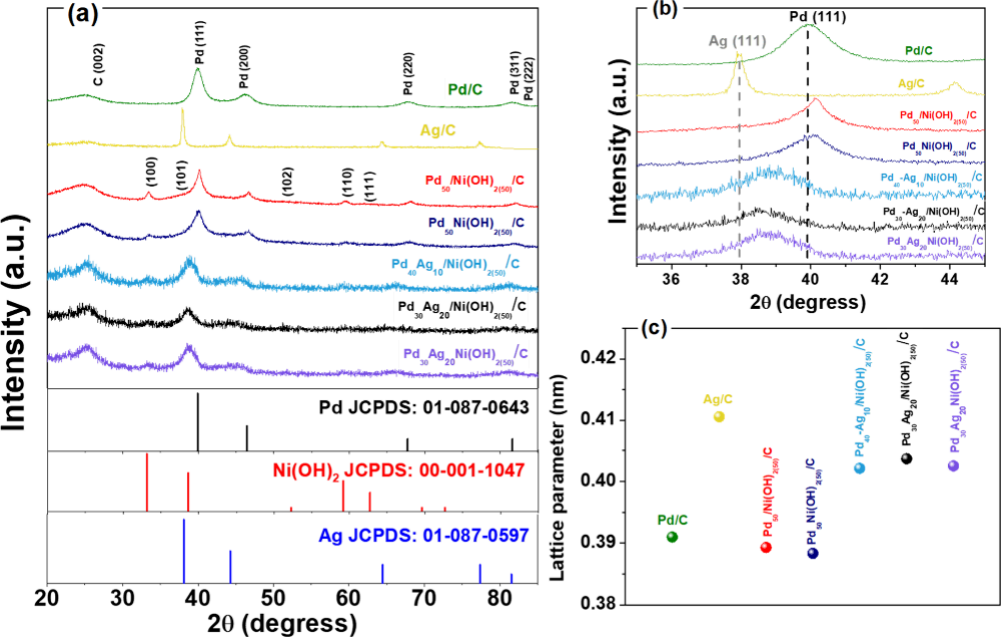
(a) X-ray diffraction of Pd/C, Ag/C, Pd_50_/Ni­(OH)_2(50)_/C, Pd_50_-Ni­(OH)_2(50)_/C, Pd_40_Ag_10_/Ni­(OH)_2(50)_/C, Pd_30_Ag_20_/Ni­(OH)_2(50)_/C, and Pd_30_Ag_20_Ni­(OH)_2(50)_/C catalysts. (b) Magnified view of the Pd(111) and Ag(111) peak regions showing peak shifts in the binary and ternary materials. (c) Lattice parameters of the various catalysts.

The other Pd_*x*_/Ni­(OH)_2(1–*x*)_/C samples in Figure S1 also display the characteristic face-centered cubic (fcc) pattern of Pd. Notably, their peaks shift to higher 2θ values relative to pure Pd, with the shift becoming more pronounced as the Ni­(OH)_2_ fraction increases. This trend is further illustrated in Figure S1b, which offers a magnified view of the (111) plane. For Pd_40_Ag_10_/Ni­(OH)_2(50)_/C, Pd_30_Ag_20_/Ni­(OH)_2(50)_/C and Pd_30_Ag_20_Ni­(OH)_2(50)_/C, the diffraction features resemble those of Pd/C, but the peak positions lie between those of elemental Pd and Ag, consistent with Pd–Ag alloy formation, as shown in the enlarged Pd(111)/Ag(111) region (Figure 1b).^6^

The average crystallite sizes for the catalysts Ag/C, Pd/C, Pd_50_/Ni­(OH)_2(50)_/C, Pd_40_Ag_10_/Ni­(OH)_2(50)_/C, and Pd_30_Ag_20_/Ni­(OH)_2(50)_/C were estimated using the Debye–Scherrer equation and are summarized in Table 1.^17,23^ The lattice parameters for the binary catalyst Pd_50_/Ni­(OH)_2(50)_/C, shown in Table 1 and illustrated in Figure 1c, are smaller than those of pure Pd. This trend becomes more pronounced with an increasing Ni content, as evidenced in Figure S1c. This suggests that the incorporation of smaller Ni atoms (135 pm) into the Pd (140 pm) crystal lattice leads to lattice contraction and may indicate potential alloy formation during the synthesis of the catalyst.^17^

**Table 1:** Physical Parameters Derived from XRD (Average Crystallite Sizes, and Lattice Parameters) and TEM Analysis (average Particle Sizes)

**catalyst**	**average particle size (nm)**	**crystallite size (nm)**	**lattice parameter (nm)**
Pd/C	3.827 ± 0.589	4.42	0.3910
Ag/C	19.748 ± 2.997	20.7	0.4106
Pd_50_/Ni(OH)_2(50)_/C	8.471 ± 3.662	9.46	0.3893
Pd_50_Ni(OH)_2(50)_/C	4.878 ± 1.048	5.52	0.3886
Pd_40_Ag_10_/Ni(OH)_2(50)_/C	4.961 ± 1.132	5.15	0.4021
Pd_30_Ag_20_/Ni(OH)_2(50)_/C	5.145 ± 1.033	5.42	0.4037
Pd_30_Ag_20_Ni(OH)_2(50)_/C	4.685 ± 1.006	4.92	0.4025

In contrast, the lattice constants of Pd_40_Ag_10_/Ni­(OH)_2(50)_/C, Pd_30_Ag_20_/Ni­(OH)_2(50)_/C and Pd_30_Ag_20_Ni­(OH)_2(50)_/C exceed those of Pd/C but remain below those of Ag/C, consistent with lattice expansion from substitution of Pd by the larger Ag atoms (144 pm).^6^ Notably, catalysts prepared by simultaneous reduction (Pd_50_Ni­(OH)_2(50)_/C and Pd_30_Ag_20_Ni­(OH)_2(50)_/C) exhibit smaller lattice parameters than their sequentially reduced counterparts. These outcomes indicate that the preparation method influences the crystal structure of Pd and suggest a higher degree of alloying achieved through the simultaneous reduction method.

Figure 2 displays TEM micrographs and the corresponding particle-size distribution histograms for the following catalysts: binary Pd_50_/Ni­(OH)_2(50)_/C, Pd_50_Ni­(OH)_2(50)_/C, and ternary Pd_30_Ag_20_/Ni­(OH)_2(50)_/C, Pd_30_Ag_20_Ni­(OH)_2(50)_/C. In Figure 2a, the nanoparticles of the Pd_50_/Ni­(OH)_2(50)_/C catalyst appear agglomerated and spherical, with diameters ranging from 4 to 16 nm, as shown in Figure 2b. In contrast, the TEM image for the Pd_50_Ni­(OH)_2(50)_/C catalyst (Figure 2c) exhibits better dispersed nanoparticles on the carbon support, with sizes approximately between 3 and 7 nm, further detailed in the histograms in Figure 2d and Table 1. The ternary catalysts exhibit a similar trend. The Pd_30_Ag_20_Ni­(OH)_2(50)_/C catalyst (Figure 2g,h) shows smaller and more dispersed nanoparticles compared to Pd_30_Ag_20_/Ni­(OH)_2(50)_/C (Figure 2e,f) and Pd_40_Ag_10_Ni­(OH)_2(50)_/C (Figure S2a,b).

**Figure 2:**
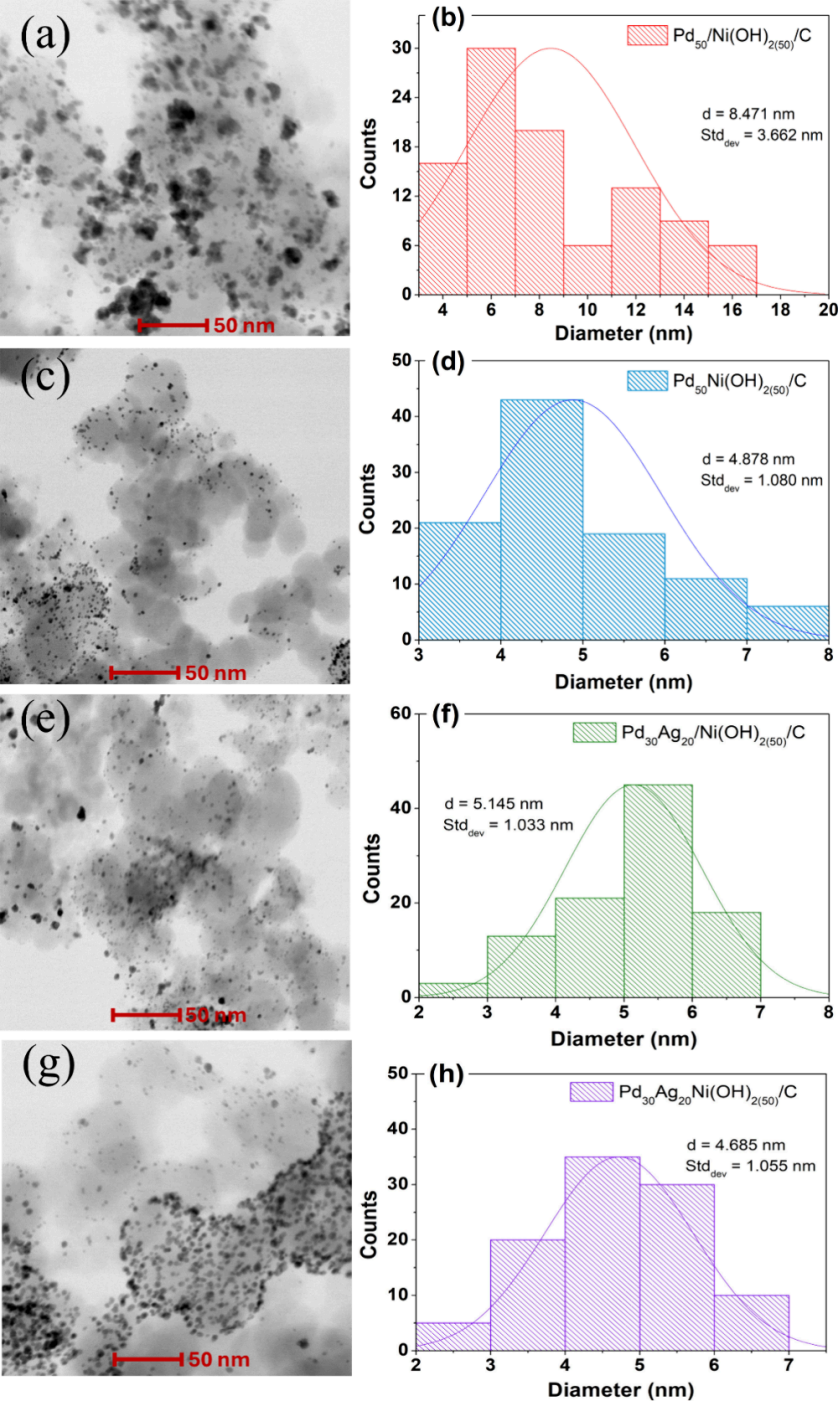
(a, c, e, g) TEM images and (b, d, f, h) histograms showing the average particle size distribution (*d*_m_) and standard deviation (Stdev) for the catalysts Pd_50_/Ni­(OH)_2(50)_/C, Pd_50_Ni­(OH)_2(50)_/C, Pd_30_Ag_20_/Ni­(OH)_2(50)_/C, and Pd_30_Ag_20_Ni­(OH)_2(50)_/C. These histograms were derived from the analysis of 100 nanoparticles to estimate the average particle size.

Analysis of the TEM images and particle size distribution for the monometallic Pd/C (Figure S2c,d) and Ag/C (Figure S2e,f) catalysts reveals that Pd nanoparticles are smaller and more uniformly dispersed on the carbon support compared to Ag nanoparticles. A similar trend of increasing particle size with higher Ag content was observed in the ternary catalysts prepared via the sequential reduction method. Notably, the ternary catalyst synthesized by the simultaneous reduction route (Pd_30_Ag_20_Ni­(OH)_2(50)_/C) exhibited smaller and more uniformly distributed nanoparticles than its sequentially prepared counterparts. This suggests that the order of precursor addition plays a significant role in modulating the nucleation and growth dynamics of Pd, Ni, and Ag nanoparticles.

The improved dispersion observed in the simultaneously reduced catalyst may be attributed to the presence of Ni^2+^ ions, which can act as a mild reducing agent due to their relatively more negative standard reduction potential. This can facilitate the coreduction of Pd^2+^ and Ag^+^ ions, promoting a more controlled nucleation process and uniform metal distribution.^13,23^ Additionally, EDX analysis (Figure S3 and Table S1) confirmed the presence of Pd, Ag, and Ni in the synthesized catalysts, with elemental compositions closely matching the nominal values.

Comparison of the Pd K edge XANES in Figure 3a shows no significant shift, suggesting no substantial change in the oxidation state of palladium, both showing good agreement with the Pd reference foil (Figures S4 and S5). A marked reduction in oscillation amplitude is observed for Pd_50_ *-*Ni­(OH)_2(50)_/C relative to Pd_50_/Ni­(OH)_2(50)_/C; the absorption oscillations in Pd_50_ *-*Ni­(OH)_2(50)_/C are substantially smaller. This translates into a drop in the intensity of the oscillations in the *R* space (Figure 3b), apart from an increase in intensity at low radial distances (Table 2). This would suggest that Pd_50_-Ni­(OH)_2(50)_/C is also coordinated to a low-*Z* element, most likely oxygen. Apart from this, the *R* space is consistent in both materials with that of a face-centered cubic palladium.

**Figure 3:**
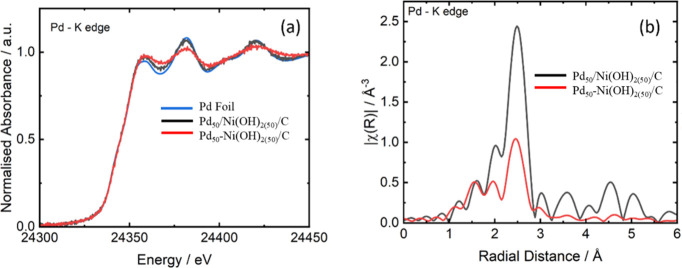
(a) Normalized Pd K-edge XANES spectra and (b) Fourier transforms of EXAFS for a Pd foil reference and the catalysts (Pd_50_/Ni­(OH)_2(50)_/C) and (Pd_50_-Ni­(OH)_2(50)_/C).

**Table 2:** Structural Parameters from EXAFS Fits at the Pd K Edge for Pelletized Catalysts: Bond Lengths, Debye–Waller Factors (DW), and Coordination Numbers (CN)^a^

**material**	**shell**	distance/Å	**DW/Å** ^**2**^	**CN**	*R*-factor
Pd_50_/Ni(OH)_2(50)_/C	Pd–Pd	2.739(2)	0.0057(3)	10.5(6)	0.006
Pd_50_-Ni(OH)_2(50)_/C	Pd–O	1.97(2)	0.004(5)	1.2(6)	0.02
	Pd–Pd	2.718(7)	0.001(1)	6.5(9)	

a The amplitude reduction factor was fixed at 0.78, and the energy shift was refined to −5(1) eV for all samples.

The first shell of Pd_50_/Ni­(OH)_2(50)_/C was fit with a single Pd–Pd scattering path, while Pd_50_ *-*Ni­(OH)_2(50)_/C was fit with two shells, the first as a Pd–O scattering path and the second as a Pd–Pd scattering path. The main difference observed between the two materials is a drop in the Pd–Pd coordination number from 10.5(6) in Pd_50_/Ni­(OH)_2(50)_/C, going down to 6.5(9) in Pd_50_ *-*Ni­(OH)_2(50)_/C. This reduction in the coordination number is likely a result of the difference in Pd particle size, which aligns with the average particle size distribution (Figure 2b,d). If the Pd particle size is reduced, there will be a higher abundance of lower coordinate surface Pd atoms compared to the bulk, fully coordinated atoms. This would also explain the presence of Pd–O scattering, as the surface of the palladium particles is likely covered in a surface layer of oxygen from being exposed to air. When the particle size is smaller, the proportion of this oxide layer is bigger, making it detectable in the EXAFS. This also suggests that the Pd_50_/Ni­(OH)_2(50)_/C catalyst exhibits a lower density of Pd–Pd neighbors, consistent with partial incorporation of Ni into the Pd lattice. This structural modification provides further evidence of alloy formation.

Unfortunately, XAS data were not obtained for Pd_30_Ag_20_/Ni­(OH)_2(50)_/C and Pd_30_Ag_20_Ni­(OH)_2(50)_/C catalysts as the Pd K (24.35 keV) and Ag K (25.5 keV) edges overlap, making the EXAFS data uninterpretable, especially for metal–metal coordination. Additionally, the similar masses of Pd (106.4 g mol^–1^) and Ag (107.9 g mol^–1^) make it impossible to distinguish between them. Thus, EXAFS is not an appropriate technique to examine Pd–Ag alloying.

The XANES at the Ni K edge was measured for both Pd_50_/Ni­(OH)_2(50)_/C and Pd_50_-Ni­(OH)_2(50)_/C (Figures S6 and S7). The absorbance from Pd_50_-Ni­(OH)_2(50)_/C is much higher compared to Pd_50_/Ni­(OH)_2(50)_/C (Figure 4), likely due to a much lower presence of Ni in this sample. This also means that the XANES and EXAFS contain too much noise to be analyzed further in Pd_50_/Ni­(OH)_2(50)_/C; therefore, the following will focus on Pd_50_-Ni­(OH)_2(50)_/C. Comparison of the XANES with the Ni foil and Ni­(OH)_2_ shows that the major structure present is Ni­(OH)_2_. There are, however, differences in the XANES prepeak between the standard and the sample, which can be explained by the presence of a small fraction of nickel metal contributing.

**Figure 4:**
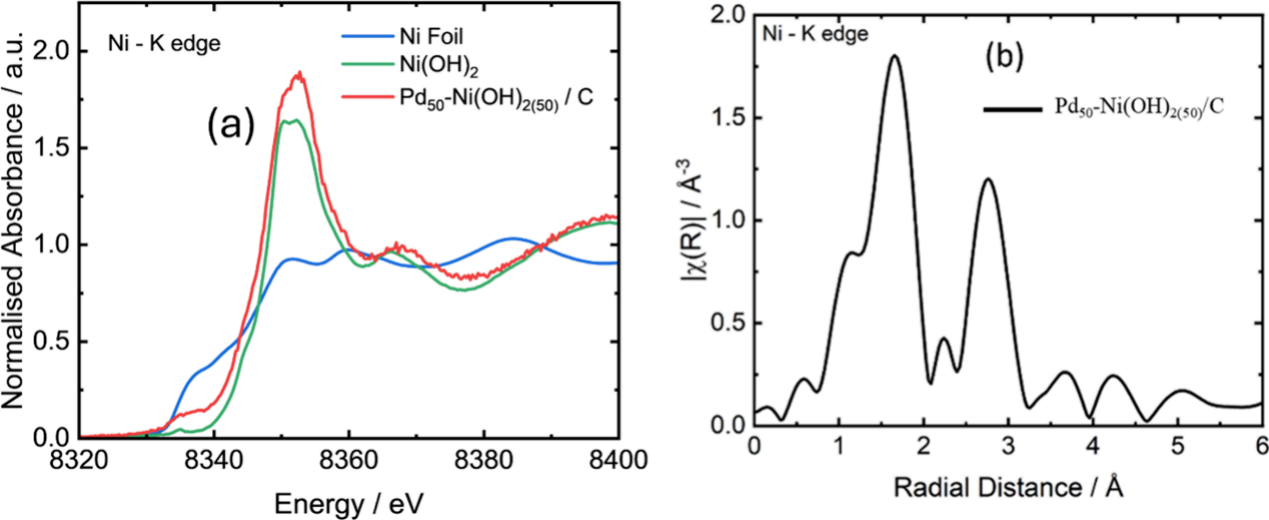
(a) Normalized Ni K-edge XANES spectra and (b) Fourier transforms of EXAFS for the Pd_50_-Ni­(OH)_2(50)_/C catalysts. For comparison, Ni foil and Ni­(OH)_2_ standard XANES spectra are included in (a). The Ni­(OH)_2_ reference spectrum was obtained from the MDR XAFS Database, specifically from the data set “XAFS spectrum of nickel hydroxide” by Ishii, M., Industrial Application and Partnership Division, DOI: 10.48505/nims.2939, published by SPring-8, and available under a Creative Commons Attribution 4.0 International License.^24–26^

The EXAFS data were fitted between 1 and 3 Å. The model comprised two coordination shells: a Ni–O first shell and a Ni–Ni second shell (Table 3), consistent with the Ni­(OH)_2_ structure. The coordination numbers were fixed at their nominal values for bulk Ni­(OH)_2_, as allowing them to vary did not result in any improvement in the model.

**Table 3:** Ni-Centered Bond Lengths and Debye–Waller Factors (DW) Obtained from EXAFS Fits for the Pd_50_-Ni­(OH)_2(50)_/C Catalyst Pellet (Pd_50_/Ni­(OH)_2(50)_/C NCs)^a^

**material**	**shell**	distance/Å	**DW/Å** ^**2**^	**CN**	*R*-factor
Pd_50_-Ni(OH)_2(50)_/C	Ni–O	2.05(2)	0.003(2)	6	0.03
	Ni–Ni	3.12(2)	0.008(2)	6	

a The amplitude reduction factor was fixed at 0.78, and the energy shift refined to 6(1) eV.

Figure S8 shows the Fourier-transformed EXAFS data for the Pd_50_/Ni­(OH)_2(50)_/C and Pd_50_-Ni­(OH)_2(50)_/C catalysts, together with reference spectra for Ni­(OH)_2_ and Pd foil. The Ni K-edge results for the Ni spectra (Figure S8a) do not show features consistent with a metallic Ni environment (either Ni-rich or Pd-rich), supporting assignment of Ni to a Ni­(OH)_2_-like local structure. The Pd K-edge R-space spectra (Figure S8b) indicate that the sequentially prepared Pd_(50)_/Ni­(OH)_2(50)_/C sample retains a structure similar to Pd metal, with reduced peak amplitudes consistent with lower first- and higher-shell coordination numbers expected for Pd nanoparticles supported on carbon. In contrast, the codeposited Pd_(50)_-Ni­(OH)_2(50)_/C sample shows a different neighbor distribution, consistent with the differences discussed elsewhere in the manuscript, and exhibits diminished higher-shell contributions relative to the sequentially prepared Pd nanoparticles.

### 3.2 Electrochemical Characterization

Figure 5 illustrates the cyclic voltammetry profiles of the synthesized catalysts in electrolyte (0.5 mol L^–1^ H_2_SO_4_) purged with CO. The ternary catalysts exhibited distinct behavior compared to the Pd/C catalyst, as indicated by the presence of several peaks during the CO oxidation process. Peak A is attributed to CO oxidation occurring at the Ag and Pd–Ag sites. This finding is further supported by the cyclic voltammograms of Ag/C shown in Figure 5b, which demonstrate the activity of Ag for CO oxidation.

**Figure 5:**
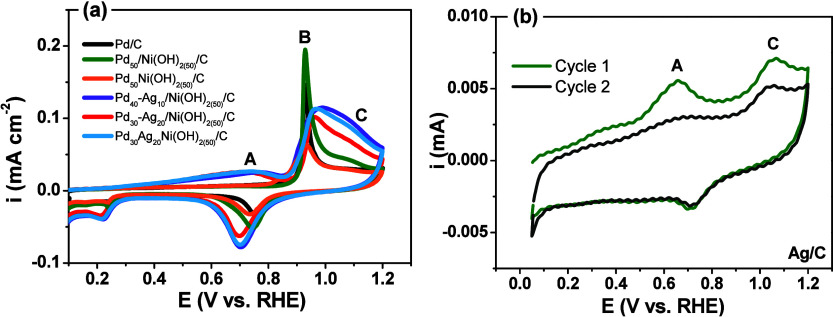
Cyclic voltammograms depicting the oxidation of a CO monolayer in 0.5 mol L^–1^ H_2_SO_4_ at a scan rate of 20 mV s^–1^: (a) for Pd/C, Pd_50_/Ni­(OH)_2(50)_/C, Pd_50_Ni­(OH)_2(50)_/C, Pd_40_Ag_10_/Ni­(OH)_2(50)_/C, Pd_30_Ag_20_/Ni­(OH)_2(50)_/C, and Pd_30_Ag_20_Ni­(OH)_2(50)_/C; (b) for Ag/C.

Peak B, indicative of CO oxidation at Pd active sites, is observed in both Pd/C and Pd_50_ */*Ni­(OH)_2(50)_/C catalysts, indicating that Ni­(OH)_2_ does not participate in CO oxidation. Finally, peak C, exclusive to the ternary catalysts Pd_40_Ag_10_/Ni­(OH)_2(50)_/C, Pd_30_Ag_20_/Ni­(OH)_2(50)_/C, and Pd_30_Ag_20_Ni­(OH)_2(50)_/C, represents an overlap of Ag oxidation and CO oxidation on the catalyst surface.

The CO oxidation onset potentials are ranked as follows: Pd_30_Ag_20_Ni­(OH)_2(50)_/C ≅ Pd_30_Ag_20_/Ni­(OH)_2(50)_/C < Pd_40_Ag_10_/Ni­(OH)_2(50)_/C < Pd_50_-Ni­(OH)_2(50)_/C < Pd_50_/Ni­(OH)_2(50)_/C ≅ Pd/C. Notably, the Pd_30_Ag_20_/Ni­(OH)_2(50)_/C catalyst shows the lowest CO-oxidation onset potential, 0.448 V, i.e., 0.482 V below that of the Pd benchmark. This superior activity of the ternary formulations is plausibly linked to Ni­(OH)_2_, which promotes water activation at lower potentials than Pd (bifunctional effect). This dissociation generates a significant amount of hydroxyl (OH) species that promote the oxidative removal of adsorbed CO and alter the electronic structure due to alloy formation between palladium (Pd) and silver (Ag).^27^

Figure 6 presents the voltammetric profiles performed in N_2_-saturated 0.5 mol L^–1^ H_2_SO_4_. In all catalysts, characteristic regions of specific interfacial reactions are identifiable. Regions A/A′ (0.05–0.25 V) correspond to the hydrogen desorption/adsorption processes on the catalyst surface. Region B, covering from 0.25 to 0.75 V, refers to the charging of the electric double layer. Region C (0.75–1.2 V) is related to the oxidation of Pd to form Pd­(II) oxide on the catalyst surface.^5^ Ternary catalysts exhibited additional features in this potential region, most notably the emergence of Peak D, which is attributed to the formation of AgOH and/or AgO surface species.^6^ The increase in peak D charge with Ag content further corroborates its association with Ag-based surface species. This assignment is consistent with the voltammetric profile of the Ag/C catalyst (Figure S9), in which anodic peaks A1 and A2 are associated with the formation of Ag_2_O and AgO, respectively, and cathodic peaks C1 and C2 with their subsequent reduction. Accordingly, the presence of peak D in the voltammograms of the ternary catalysts supports the exposure of Ag-containing sites at the electrode surface.

**Figure 6:**
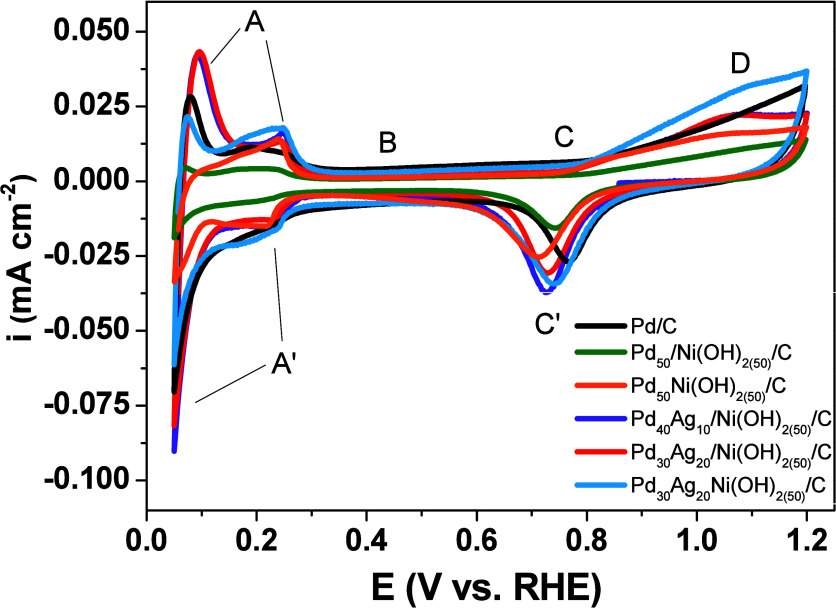
Cyclic voltammograms (second cycle) for catalysts Pd/C, Pd_50_/Ni­(OH)_2(50)_/C, Pd_50_-Ni­(OH)_2(50)_/C, Pd_40_Ag_10_/Ni­(OH)_2(50)_/C, Pd_30_Ag_20_/Ni­(OH)_2(50)_/C, and Pd_30_Ag_20_Ni­(OH)_2(50)_/C in 0.5 mol L^–1^ H_2_SO_4_ at 20 mV s^–1^. Regions A/A′, B, C/C′, and D correspond to different processes occurring on the catalyst’s surface.

Peak C′ in Figure 6 results from the reduction of Pd­(II) oxide. In binary and ternary catalysts, this peak appears at lower potentials than in the Pd/C catalyst. The change can be ascribed to altered surface binding of CO and OH^–^, driven by the higher oxophilicity of Ag and Ni­(OH)_2_, relative to Pd.^5^

The electrochemically active surface area (ECSA) was obtained by integrating the charge *Q* from the CO-stripping voltammograms. ECSA (cm^2^ mg^–1^) was then calculated as ECSA = *Q*/(0.420 × *M*_Pd_), where 0.420 mC cm^–2^ corresponds to the charge for oxidizing a CO monolayer, and *M*_Pd_ is the Pd mass in the catalyst.^1^ Table 4 presents the calculated ECSA values for the catalysts examined. The ECSA value for Pd_30_Ag_20_/Ni­(OH)_2(50)_/C was 791.3 cm^2^ mg^–1^, approximately 3.4 times greater than that of Pd/C.

**Table 4:** Electrochemical Parameters Obtained during the Oxidation of CO and Formic Acid on the Evaluated Catalysts

**catalyst**	**ECSA** **(cm** ^**2**^ **mg** ^**–1**^ **)**	***E***_**CO**_ **(V)**	***E***_**FAOR**_ **(V)**	**specific activity** **(mA cm** ^**–2**^ **)** ^a^	**mass activity** **(mA mg**_Pd_**** ^**–1**^ **)** ^b^
Pd/C	231.7	0.850	0.143	1.15	266.46
Pd_50_/Ni(OH)_2(50)_/C	395.5	0.857	0.152	1.80	1039.50
Pd_50_-Ni(OH)_2(50)_/C	577.5	0.712	0.100	3.05	1205.88
Pd_40_Ag_10_/Ni(OH)_2(50)_/C	706.1	0.475	0.133	1.36	960.29
Pd_30_Ag_20_/Ni(OH)_2(50)_/C	767.7	0.450	0.126	5.85	4491.05
Pd_30_Ag_20_Ni(OH)_2(50)_/C	791.3	0.448	0.128	7.79	6164.23

a Measured at the anodic peak.

b Mass current = specific activity × ECSA.

The catalytic behavior of the synthesized catalysts was assessed by cyclic voltammetry in 0.5 mol L^–1^ formic acid containing 0.5 mol L^–1^ H_2_SO_4_, as illustrated in Figure 7a. All catalysts exhibited two well-defined peaks, indicative of characteristic electrochemical processes and providing key insights into their catalytic behavior.

**Figure 7:**
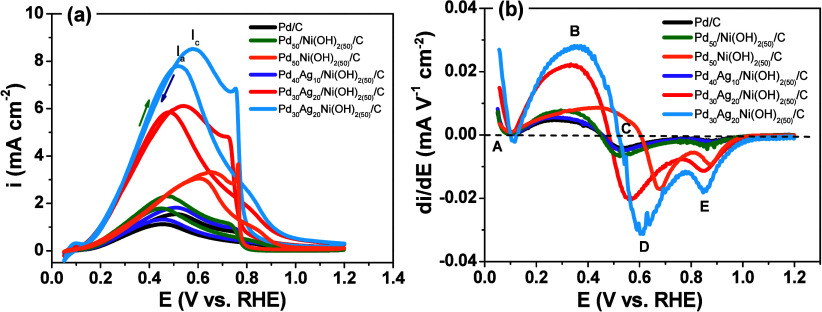
(a) Second cycle cyclic voltammograms for formic acid oxidation (0.5 mol L^–1^ in 0.5 mol L^–1^ H_2_SO_4_, 25 °C) on Pd/C, Pd_50_/Ni­(OH)_2(50)_/C, Pd_50_Ni­(OH)_2(50)_/C, Pd_40_Ag_10_/Ni­(OH)_2(50)_/C, Pd_30_Ag_20_/Ni­(OH)_2(50)_/C, and Pd_30_Ag_20_Ni­(OH)_2(50)_/C. υ = 20 mV s^–1^. Oxidation peaks are indicated as i_a_ (anodic) and i_c_ (cathodic), arrows denote scan direction: (→ anodic, ← cathodic). (b) First-derivative voltammograms of the anodic branches for formic acid oxidation. Labels (a)–(e) indicate potential reactions during formic acid oxidation.

The prominent peak observed during the anodic scan (designated as peak I_a_) is ascribed to formic acid oxidation that has been freshly adsorbed onto the catalyst’s surface. Conversely, the peak identified during the cathodic scan (noted as peak i_c_) corresponds to the oxidation of carbonaceous species, including carbon monoxide (CO) and formate ions (HCOO^–^), that remained unoxidized during the anodic scan.^28^ These residual species can accumulate on the catalyst’s surface, leading to a reduction in its catalytic efficiency and performance. Such accumulation not only hinders the reactivity of the catalyst but also poses challenges for sustained catalytic action over time.^29,30^

After adsorption, the oxidation of formic acid can occur through parallel pathways. The direct path, which is faster, involves the formation of weakly adsorbed intermediates that are active but short-lived. Formate has already been identified as an adsorbed fragment on the surface in contact with formic acid in solution (reaction 1).^31^

$${\mathrm{HCOOH}}_{\mathrm{ads}}\to {\mathrm{HCOO}}_{\mathrm{ads}}+H^{+}+e^{-}$$ 1

Formate is readily converted into CO_2_ (reaction 2):

$${\mathrm{HCOO}}_{\mathrm{ads}}\to {\mathrm{CO}}_{2}+H^{+}+e^{-}$$ 2

The indirect pathway, which is slower, involves the dehydration of formic acid:

$${\mathrm{HCOOH}}_{\mathrm{ads}}\to {\mathrm{CO}}_{\mathrm{ads}}+H_{2}O$$ 3

Strongly adsorbed intermediates, such as CO_ads_, which are oxidized at high potentials (the potential at which OH is formed at an appreciable rate), follow:

$${\mathrm{CO}}_{\mathrm{ads}}+{\mathrm{OH}}_{\mathrm{ads}}\to {\mathrm{CO}}_{2}+H^{+}+e^{-}$$ 4

The presence of Ag and Ni­(OH)_2_ plays a significant role in affecting reactions 3 and 4 by enhancing the adsorption of hydroxide ions at lower potentials. This enhancement is crucial, as it facilitates the oxidation of formic acid, enabling the reaction to occur at reduced overpotentials, as clearly illustrated in Table 4. Optimizing the concentrations of Ag and Ni­(OH)_2_ is essential, as these components can effectively regenerate the active sites of Pd by supplying a steady influx of OH^–^ species at lower potentials. This regeneration process ultimately increases the overall reaction rate and enhances catalytic efficiency.

Dong et al.^32^ synthesized a series of Pd-Ni­(OH)_2_/C catalysts with varying Pd/Ni ratios for the oxidation of formic acid. They found that Ni­(OH)_2_/C exhibited negligible catalytic activity toward this reaction. Based on these findings, the authors concluded that Pd is the catalytically active component, rather than Ni. Other researchers support this conclusion.^17,18,33^ Therefore, Ni­(OH)_2_ significantly enhances the performance of Pd catalysts for formic acid oxidation through synergistic effects. It promotes the formation of adsorbed hydroxyl species, reduces the adsorption of CO (a key poison for catalysis), and improves the electronic properties of Pd. These factors contribute to increased activity, improved CO tolerance, and enhanced catalyst stability.^17,33^

However, it is important to note that an excessive amount of Ni­(OH)_2_ or hydroxyl species can lead to blockage of the active sites on the Pd surface. This inhibition hampers the adsorption of active species, negatively affecting catalyst performance. The detrimental effects of overloading with Ni­(OH)_2_ are evidenced by the experimental results for the Pd_30_/Ni­(OH_)2(70)_/C catalyst, as presented in Figure S10.

In addition, an overly high Ag content results in fewer exposed Pd atoms on the catalyst surface, thereby decreasing overall catalytic activity. Ag is generally not very active for the electro-oxidation of formic acid, compared to Pt or Pd.^13,34^ Ag promotes the “formate pathway” (direct oxidation to CO_2_) rather than the “carboxyl pathway” (which produces poisoning CO intermediates) by promoting the adsorption of OH groups (OH_ads_) that help oxidize the CO species.^34–36^ Therefore, the Pd_30_Ag_20_Ni­(OH)_2_/C catalyst presented the most balanced scenario compared to the Pd_40_Ag_10_Ni­(OH)_2_/C catalyst; it exhibits a balance of active Pd sites, necessary for the adsorption of formic acid, and abundant Ag sites, which mitigate the formation and strong adsorption of CO-like intermediates that poison the catalyst surface.

The decrease in anodic current density observed near 0.8 V (Figure 7) for all catalysts, more pronounced for Pd_30_Ag_20_/Ni­(OH)_2(50)_/C and Pd_30_Ag_20_Ni­(OH)_2(50)_/C, is associated with the onset of Pd surface oxidation and the formation of PdOH/PdO species (Figure 6, region C). At these potentials, metallic Pd^0^ sites that are required for the direct formic acid oxidation pathway (eqs 1 and 2) become progressively covered by oxide species, leading to a marked decrease in current density.^28,30,31^ The more abrupt current decay for the ternary catalysts may reflect changes in the Pd electronic structure and surface oxophilicity that promote earlier oxide formation.^5^ This behavior would increase the blocking of Pd^0^ active sites and, consequently, make the current drop more pronounced than that observed for the other catalysts.

During the reverse scan, a new anodic peak (i_c_) appears, which is attributed to the reduction of the Pd oxide layer, restoring metallic Pd^0^ active sites and reactivating formic acid oxidation.^37,38^ The sharper peak i_c_ for Pd_30_Ag_20_/Ni­(OH)_2(50)_/C and Pd_30_Ag_20_Ni­(OH)_2(50)_/C is consistent with reports on polycrystalline Pd electrodes, where rapid oxide reduction is accompanied by pronounced oxidation currents at low-index Pd planes.^38,39^

Table 4 shows important insights about the Pd/Ni­(OH)_2_/C and Pd/C catalysts. First, when we look at their specific activity, which normalizes the current based on the surface area of the active metal, we see that adding Ni­(OH)_2_ improves Pd’s catalytic activity. Second, the mass activity, which normalizes the current based on the weight of the active metal, shows that the effect of Ni­(OH)_2_ is even stronger. This means that Ni­(OH)_2_ helps make better use of Pd in the catalyst.^25,26^ Therefore, the addition of Ni­(OH)_2_ significantly enhances the dispersion of Pd on the carbon support and boosts its formic acid oxidation performance.

Figure 7b shows that the derivative voltammograms of the studied catalysts are similar, suggesting analogous reaction mechanisms. The region between points A and B represents the adsorption and oxidation of formic acid, with the subsequent removal of hydrogen atoms, as described in reactions 1–4.^31^ Point C marks the peak oxidation of formic acid. Points D and E correspond to various parallel reactions, such as the formation of CO and/or formate. The lowest onset potential for oxidation in the Pd_30_Ag_20_Ni­(OH)_2(50)_/C catalyst indicates that formic acid can be more readily oxidized electrochemically on this catalyst’s surface.^40^

Moreover, although direct comparisons across different studies are inherently limited due to variations in experimental parameters such as electrolyte composition, scan rate, and electrode configuration, a comparative analysis was conducted to contextualize the performance of the Pd_30_Ag_20_Ni­(OH)_2(50)_/C catalyst. As presented in Table 5, this ternary catalyst exhibited superior activity relative to most Pd-based systems reported in the literature. Specifically, it outperformed binary catalysts such as Pd_0.3_Cu_1_ (6.11 mA cm^–2^)^41^ and Pd/FeP (1540 mA mg_Pd_^–1^),^1^ achieving a specific activity of 7.79 mA cm^–2^ and a remarkable mass activity of 6164.23 mA mg_Pd_^–1^ under the conditions used in this work. These results highlight the enhanced electrocatalytic performance of the Pd_30_Ag_20_Ni­(OH)_2(50)_/C composite, which may be attributed to synergistic effects among its metallic components and the presence of Ni­(OH)_2_ promoting hydroxyl species adsorption.

**Table 5:** Comparison of Studies on Pd-based Catalysts for Formic Acid Oxidation

**catalyst**	**supporting electrolyte**	**FA concentration** **(mol L** ^**–1**^ **)**	**scan rate** **(mV s** ^**–1**^ **)**	**specific activity** **(mA cm** ^**–2**^ **)**	**ref**
ULT-Pd	0.5 M H_2_SO_4_	0.5	50	0.74	^42^
Pt/Pd	0.5 M H_2_SO_4_	0.25	50	1.25	^43^
PdAg	0.1 M HClO_4_	0.5	100	3.82	^6^
Pd_0.3_Cu_1_	0.5 M H_2_SO_4_	0.5	50	6.11	^41^
PdNi/C	0.5 M H_2_SO_4_	0.5	50	2.76	^31^
Pd/FeP	0.5 M H_2_SO_4_	0.5	50	1540^a^	^1^
Pd_1_Ni_1_-NNS/RGO	0.5 M H_2_SO_4_	0.5	50	604.3^a^	^44^
PdSnAg/C	0.5 M H_2_SO_4_	0.5	50	629.9^a^	^45^
PdNiCu/C	0.5 M H_2_SO_4_	0.5	20	792^a^	^46^
Au@AuPd NCs	0.5 M H_2_SO_4_	0.5	50	1250^a^	^47^
Pd_30_Ag_20_Ni(OH)_2(50)_/C	0.5 M H_2_SO_4_	0.5	20	7.79 (6164.2)^a^	this paper

a These values are expressed in terms of mass activity (mA mg_Pd_^–1^).

Figure S11 shows the cyclic voltammograms for the catalysts Pd/C, Pd_50_Ni­(OH)_2(50)_/C, and Pd_30_Ag_20_Ni­(OH)_2(50)_/C in 0.5 mol L^–1^ H_2_SO_4_ and 0.5 mol L^–1^ formic acid solutions at different scan rates. For all catalysts, the maximum current density for formic acid oxidation increases with scan rate. The current densities at peak i_a_ are linearly proportional to the square root of the scan rate, suggesting that the electrocatalytic oxidation of formic acid in all the catalysts studied can be controlled by diffusion.^48,49^

Chronoamperometric measurements were subsequently performed to evaluate the stability of the Pd_30_Ag_20_Ni­(OH)_2(50)_/C catalyst (Figure 8). Under identical conditions, the ternary catalyst sustained current densities more than three times higher than the Pd/C reference catalyst (0.0831 mA cm^–2^), confirming its superior resistance to deactivation. Furthermore, cyclic voltammograms recorded before and after the extended chronoamperometric tests (Figure 8b,c) showed only a slight decrease in current density for both electrodes, with Pd_30_Ag_20_Ni­(OH)_2(50)_/C maintaining a significantly higher electrochemical response than Pd/C. Therefore, these results demonstrate that the incorporation of Ag and Ni­(OH)_2_ enhances the long-term stability of the catalyst without compromising its activity under extended operation.

**Figure 8:**
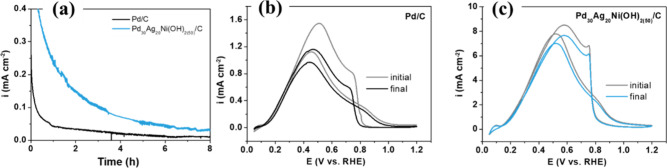
(a) Long-term chronoamperometry for Pd/C and Pd_30_Ag_20_Ni­(OH)_2(50)_/C at 0.2 V vs RHE for 8 h in 0.5 mol L^–1^ H_2_SO_4_. (b, c) Second cycle cyclic voltammograms before and after the 8 h chronoamperometric test for Pd/C (b), and Pd_30_Ag_20_Ni­(OH)_2(50)_/C (c) in 0.5 mol L^–1^ H_2_SO_4_ at a scan rate of 20 mV s^–1^.

Figure S12 presents a TEM image, the average particle size distribution, and the chemical composition of the Pd_30_Ag_20_Ni­(OH)_2(50)_/C catalyst after conducting a stability test. The TEM image (Figure S12a) reveals particle agglomeration, likely caused by the dissolution of smaller particles, which then redeposit onto larger nanoparticles.^50^ This process results in an overall increase in the average particle size, as illustrated in the nanoparticle histogram (Figure S12b). In addition, there is an observed increase in Pd concentration and a decrease in Ni and Ag concentrations (Figure S12c), suggesting that Ni and Ag dissolved during the long-term durability test. However, as shown in Figure 8, this change has a negligible effect on the material’s catalytic activity.

## 4 Conclusions

This work demonstrates that the synthesis sequence exerts a decisive influence on the surface and electronic structure of Pd–Ag–Ni­(OH)_2_ electrocatalysts and, consequently, on their FAOR performance in acidic media. The catalysts that were produced via simultaneous reduction displayed smaller, well-dispersed nanoparticles on carbon and a higher degree of alloying between catalytic species than those prepared by sequential reduction, as observed by XANES/EXAFS experiments. This modification of the catalyst structure was paralleled directly by an enhanced electrocatalytic activity that was observed for the oxidation of formic acid. Specifically, the catalysts containing up to 20% Ag displayed superior electrocatalytic performance toward formic acid oxidation than the Pd/C catalyst. This higher activity likely stems from easier adsorption of oxygenated species at Ag sites, which in turn promotes the oxidation of formic acid on adjacent Pd ensembles. In addition, the Pd_30_Ag_20_Ni­(OH)_2(50)_/C catalyst exhibits excellent stability during formic acid oxidation, indicating that Ni­(OH)_2_ and Ag help stabilize Pd nanoparticles and mitigate poisoning by adsorbed intermediates. Moreover, the Pd_30_Ag_20_Ni­(OH)_2(50)_/C catalyst is more active than several other materials documented in the literature, thereby making it a promising candidate for use in direct formic acid fuel cells. Overall, our results demonstrate that controlling synthesis sequence is an effective route to tailor the surface electronic structure, nanostructure, and catalytic properties of Pd-based multicomponent materials, in line with the broader processing-structure–property paradigm in functional materials.

## Supplementary Material



## Notes

Experimental details for physical characterization (XRD, TEM, TEM-EDX, XAFS) and electrochemical measurements (cell setup, ink preparation, activation protocols, CO stripping, FAOR tests, and specific activity calculations); XRD patterns of Pd/C and Pd_*x*_/Ni­(OH)_2__(*y*)_/C catalysts, including a magnified Pd(111) region and lattice parameters (Figure S1); TEM images and particle size distribution for Pd_40_Ag_10_Ni­(OH)_2(50)_/C, Pd/C, and Ag/C catalysts (Figure S2); TEM-EDX chemical compositions and elemental summaries (Pd, Ni, Ag) for all catalysts (Figure S3 and Table S1); Pd and Ni K-edge XAFS data, including *k* ^2^-weighted FT signals, *k*-space plots, and Ni K-edge XANES spectra for Pd_50_/Ni­(OH)_2(50)_/C and Pd_50_Ni­(OH)_2(50)_/C (Figures S4–S7); additional references related to Ni­(OH)_2_-supported catalysts and XAFS data analysis (Figure S8); cyclic voltammograms for Ag/C in 0.5 mol L^–1^ H_2_SO_4_ with assignment of Ag_2_O/AgO peaks (Figure S9); FAOR voltammograms for Pd/C and Pd_*x*_/Ni­(OH)_2__(1–*x*)_/C catalysts with identification of anodic and cathodic peaks (Figure S10); cyclic voltammograms at different scan rates for Pd/C, Pd_50_Ni­(OH)_2(50)_/C and Pd_30_Ag_20_Ni­(OH)_2(50)_/C (Figure S11); and TEM image and TEM-EDX chemical composition of Pd_30_Ag_20_Ni­(OH)_2(50)_/C after stability test (Figure S12) (PDF)

## References

[1] Bao Y., Liu H., Liu Z., Wang F., Feng L. (2020). Pd/FeP catalyst engineering
via thermal annealing for improved formic acid electrochemical oxidation. Appl. Catal. B: Environ..

[2] Antolini E. (2009). Palladium
in fuel cell catalysis. Energy Environ. Sci..

[3] Salazar-Banda G. R., Suffredini H. B., Avaca L. A., Machado S. A. S. (2009). Methanol and
ethanol electro-oxidation on Pt–SnO_2_ and Pt–Ta_2_O_5_ sol–gel-modified boron-doped diamond
surfaces. Mater. Chem. Phys..

[4] Li R., Lin J., Zhang H., Gong Y., Zhang X., Yu C., Tang C., Huang Y. (2025). Improving electrocatalytic formic
acid oxidation performance via catalyst layer design utilizing porous
boron nitride-supported Pd catalysts. J. Power
Sources.

[5] Almeida C. V. S., Eguiluz K. I. B., Salazar-Banda G. R. (2020). Superior
ethanol electrooxidation activity of Pd supported on Ni­(OH)_2_/C: The effect of Ni­(OH)­2 nanosheets content. J. Electroanal. Chem..

[6] Fu G. T., Liu C., Zhang Q., Chen Y., Tang Y. W. (2015). Polyhedral palladium–silver
alloy nanocrystals as highly active and stable electrocatalysts for
the formic acid oxidation reaction. Sci. Rep..

[7] Hammer B., Nørskov J. K. (2000). Theoretical surface science and catalysis—calculations
and concepts. Adv. Catal..

[8] Zhang G., Shi Y., Fang Y., Cao D., Guo S., Wang Q., Chen Y., Cui P., Cheng S. (2021). Ordered PdCu-based
core–shell concave nanocubes enclosed by high-index facets
for ethanol electrooxidation. ACS Appl. Mater.
Interfaces.

[9] Goswami C., Saikia H., Tada K., Tanaka S., Sudarsanam P., Bhargava S. K., Bharali P. (2020). Bimetallic palladium–nickel
nanoparticles anchored on carbon as high performance electrocatalysts
for oxygen reduction and formic acid oxidation reactions. ACS Appl. Energy Mater..

[10] Xie Y., Li D., Yang Y., Wang S., Feng L. (2025). Cerium partial fluoridation
efficiently promotes Pd nanoparticle catalysis of formic acid oxidation. Chem. Commun..

[11] Chen D., Pei S., He Z., Shao H., Wang J., Wang K., Wang Y., Jin Y. (2020). Highly active PdSn binary alloyed
catalysts supported on B- and N-codoped graphene for formic acid electro-oxidation. Catalysts.

[12] Rajesh D., Mahendiran C., Suresh C. (2020). The Promotional Effect of Ag in Pd-Ag/Carbon
Nanotube-Graphene Electrocatalysts for Alcohol and Formic Acid Oxidation
Reactions. ChemElectroChem.

[13] Karatok M., Ngan H. T., Jia X., O’Connor C. R., Boscoboinik J. A., Stacchiola D. J., Sautet P., Madix R. J. (2023). Achieving
Ultra-High Selectivity to Hydrogen Production from Formic Acid on
Pd–Ag Alloys. J. Am. Chem. Soc..

[14] Li Y.-H., Deng H.-C., Zhou Z.-H., Yang P.-P., Fei J.-J., Xie Y.-X. (2023). Pd_12_Ag_1_ nanoalloy on dendritic
CNFs catalyst for boosting formic acid oxidation. Appl. Surf. Sci..

[15] Mondal S., De S. K., Ghosh T., Mondal S., Manna M., Senapati D. (2025). Pd-Lined Strained Trimetallic Au-Ag-Pd
Nanoprism for
Enhanced Electrocatalytic Activity Towards Formic Acid Oxidation. Small Sci..

[16] Weng Y.-C., Chiang C.-L. (2023). Optimization of
Pd–Co–Cu and Pd–Co–Au
catalysts for the oxidation of formic acid using scanning electrochemical
microscopy. J. Alloys Compd..

[17] Yang H., Zhang A., Bai Y., Chu M., Li H., Liu Y., Zhu P., Chen X., Deng C., Yuan X. (2022). One stone
two birds: Unlocking the synergy between amorphous Ni­(OH)_2_ and Pd nanocrystals toward ethanol and formic acid oxidation. Inorg. Chem..

[18] Sofian M., Nasim F., Ali H., Kanodarwala F. K., Nadeem M. A. (2024). Efficient formic acid oxidation over gallium oxide
incorporated Pd-containing electrocatalyst. Int. J. Hydrogen Energy.

[19] Wen X., Nazemi S. A., da Silva R. R., Moth-Poulsen K. (2023). The Effect
of the Pd Precursors on the Shape of Hollow Ag–Pd Alloy Nanoparticles
Using Ag Nanocubes as Seed. Langmuir.

[20] Li Q., Cai Q., Li X., Han E., Sun Y., Lu Y., Cai Z., Yu H. (2023). Effects of
Palladium Precursors on the Activity of
Palladium Nanocatalysts for the Oxidation of Volatile Organic Components. Nanomaterials.

[21] Chen Y., Zohaib A., Sun H., Sun S. (2025). Multi-metallic nanoparticles:
synthesis and their catalytic applications. Chem. Commun..

[22] Ricciardulli T., Gorthy S., Adams J. S., Thompson C., Karim A. M., Neurock M., Flaherty D. W. (2021). Effect of Pd Coordination and Isolation
on the Catalytic Reduction of O_2_ to H_2_O_2_ over PdAu Bimetallic Nanoparticles. J. Am. Chem. Soc..

[23] Chen J., Yang M., Pang M., Gao F., Guo P. (2021). Bimetallic
PdAg nanoparticles for enhanced electrocatalysis of ethanol oxidation
reaction. Colloids Surf., A.

[24] Ishii, M. ; Nagao, H. ; Tanabe, K. ; Matsuda, A. ; Yoshikawa, H. MDR XAFS DB. Materials Data Repository, National Institute for Materials Science, 2021.

[25] Ishii M., Tanabe K., Matsuda A., Ofuchi H., Matsumoto T., Yaji T., Inada Y., Nitani H., Kimura M., Asakura K. (2023). Integration of X-ray absorption fine
structure databases
for data-driven materials science. Sci. Technol.
Adv. Mater.: Methods.

[26] Ishii, M. ; Industrial Application and Partnership Division. XAFS Spectrum of Nickel Hydroxide. SPring-8, 2021.

[27] Wang H., Xi C., Yin H., Ding Y. (2024). Designing nanoporous Pd catalyst
with dual enhancement of thermodynamics and kinetics for formic acid
oxidation reaction. J. Alloys Compd..

[28] Li H., Xin Q., Li W., Zhou Z., Jiang L., Yang S., Sun G. (2004). An improved
palladium-based DMFCs cathode catalyst. Chem.
Commun..

[29] Obradovic M. D., Stancic Z. M., Lacnjevac U. C., Radmilovic V. V., Gavrilovic-Wohlmuther A., Radmilovic V. R., Gojkovic S. Lj. (2016). Electrochemical oxidation of ethanol on palladium–nickel
nanocatalyst in alkaline media. Appl. Catal.
B: Environ..

[30] Mota-Lima A., Gonzalez E. R., Eiswirth M. (2014). Complex electrooxidation of formic
acid on palladium. J. Braz. Chem. Soc..

[31] Chen Z., Zhang J., Zhang Y., Liu Y., Han X., Zhong C., Hu W., Deng Y. (2017). NiO-induced
synthesis
of PdNi bimetallic hollow nanocrystals with enhanced electrocatalytic
activities toward ethanol and formic acid oxidation. Nano Energy.

[32] Dong Y., Chen Q., Qiu C., Ma X., Wang Y., Sun T., Fan G. (2020). Synergistic catalysis
of Pd-Ni­(OH)_2_ hybrid
anchored on porous carbon for hydrogen evolution from the dehydrogenation
of formic acid. Int. J. Hydrogen Energy.

[33] Jiang Y., Chen J., Zhang J., Zeng Y., Wang Y., Zhou F., Kiani M., Wang R. (2017). Controlled decoration
of Pd on Ni­(OH)_2_ nanoparticles by atomic layer deposition
for high ethanol oxidation activity. Appl. Surf.
Sci..

[34] Liu D., Xie M., Wang C. (2016). Pd-Ag alloy hollow nanostructures with interatomic
charge polarization for enhanced electrocatalytic formic acid oxidation. Nano Res..

[35] Podlovchenko B. I., Maksimov Y. M., Gladysheva T. D. (2023). The effect of small
silver inclusions on the palladium activity in formic acid oxidation
reaction and corrosion stability. J. Solid State
Electrochem..

[36] Sneka-Płatek O., Kaźmierczak K., Jędrzejczyk M., Sautet P., Keller N., Michel C., Ruppert A. M. (2020). Understanding the influence of the
composition of the Ag single bond Pd catalysts on the selective formic
acid decomposition and subsequent levulinic acid hydrogenation. Int. J. Hydrogen Energy..

[37] Capon A. (1973). The oxidation
of formic acid at noble metal electrodes. J.
Electroanal. Chem..

[38] Hoshi N., Kida K., Nakamura M., Nakada M., Osada K. (2006). Structural
Effects of Electrochemical Oxidation of Formic Acid on Single Crystal
Electrodes of Palladium. J. Phys. Chem. B.

[39] Manzanares M. I., Pavese A. G., Solis V. M. (1991). Comparative investigation of formic
acid and formaldehyde electrooxidation on palladium in acidic medium.
Effect of surface oxides. J. Electroanal. Chem. Interf. Electrochem..

[40] Almeida C. V. S., Ferreira D. S., Huang H., Gaiotti A. C., Camara G. A., Russell A. E., Eguiluz K. I. B., Salazar-Banda G. R. (2019). Highly
active Pt_3_Rh/C nanoparticles towards ethanol electrooxidation:
Influence of the catalyst structure. Appl. Catal.
B: Environ..

[41] Tai X., Wu B., Bao J., Qu W., Zhao L., Wang Z. (2022). Galvanic replacement-mediated
synthesis of Pd–Cu alloy nanospheres as electrocatalysts for
formic acid oxidation. Mater. Today Sustain..

[42] Gharib A., Arab A. (2021). Improved formic acid
oxidation using electrodeposited Pd–Cd
electrocatalysts in sulfuric acid solution. Int. J. Hydrogen Energy.

[43] Zhu F., Ma G., Bai Z., Hang R., Tang B., Zhang Z., Wang X. (2013). High activity
of carbon nanotubes supported binary and ternary Pd-based
catalysts for methanol, ethanol and formic acid electro-oxidation. J. Power Sources.

[44] Bin D., Yang B., Ren F., Zhang K., Yang P., Du Y. (2015). Facile synthesis of
PdNi nanowire networks supported on reduced graphene
oxide with enhanced catalytic performance for formic acid oxidation. J. Mater. Chem. A.

[45] Zhu F., Wang M., He Y., Ma G., Zhang Z., Wang X. (2014). A comparative study of elemental additives (Ni, Co, and Ag) on electrocatalytic
activity improvement of PdSn-based catalysts for ethanol and formic
acid electro-oxidation. Electrochim. Acta.

[46] Hu S., Munoz F., Noborikawa J., Haan J., Scudiero L., Ha S. (2016). Carbon supported Pd-based
bimetallic and trimetallic catalysts for
formic acid electrochemical oxidation. Appl.
Catal. B: Environ..

[47] Li D.-N., Wang A.-J., Wei J., Zhang Q.-L., Feng J.-J. (2017). Facile
synthesis of flower-like Au@AuPd nanocrystals with highly electrocatalytic
activity for formic acid oxidation and hydrogen evolution reactions. Int. J. Hydrogen Energy.

[48] Marinšek M., Šala M., Jančar B. (2013). A study towards superior carbon nanotubes-supported
Pd-based catalysts for formic acid electro-oxidation: Preparation,
properties and characterisation. J. Power Sources.

[49] Wang Y., Wu B., Gao Y., Tang Y., Lu T., Xing W., Liu C. (2009). Kinetic study
of formic acid oxidation on carbon supported Pd electrocatalyst. J. Power Sources.

[50] Sneed B. T., Young A. P., Jalalpoor D., Golden M. C., Mao S., Jiang Y., Wang Y., Tsung C.-K. (2014). Shaped Pd–Ni–Pt
Core–Sandwich–Shell Nanoparticles: Influence of Ni Sandwich
Layers on Catalytic Electrooxidations. ACS Nano.

