# Supplementary material for: Synthesis-Sequence-Controlled Surface and Electronic Structure in Pd–Ag–Ni(OH)2/C Electrocatalysts for Efficient Formic Acid Oxidation
Source: ACS Omega. 2026 May 12;11(20):29603–13. doi: 10.1021/acsomega.5c12585 (PMC13216916; doi:10.1021/acsomega.5c12585)
Supplement: Supplementary file 1 [file ao5c12585_si_001.pdf]

Supporting Information for the paper

Synthesis-Sequence–Controlled Surface and Electronic Structure in Pd–Ag–Ni(OH)$_2$/C Electrocatalysts for Efficient Formic Acid Oxidation

Maria E. S. C. Argôlo[a,b], Caio V. S. Almeida[a,c], Connor Sherwin[d], Andrea E. Russell[d], Katlin I. B. Eguiluz[a,c,] Giancarlo R. Salazar-Banda[a,c,*]*

[a] Laboratory of Electrochemistry and Nanotechnology, Institute of Technology and Research, 49.032-490, Aracaju, Sergipe, Brazil.

[b] Chemical Engineering Department, Tiradentes University, 49.032-490, Aracaju, Sergipe, Brazil.

[c] Process Engineering Postgraduate Program, Tiradentes University, 49.032-490, Aracaju, Sergipe, Brazil.

[d] School of Chemistry, University of Southampton, University Road, Southampton SO17 1BJ, United Kingdom.

*Corresponding author: katlinbarrios@gmail.com (Katlin Ivon Barrios Eguiluz)

*Corresponding author: gianrsb@gmail.com (Giancarlo Richard Salazar-Banda)

Experimental Details

Physical characterization

The crystalline structures of the catalysts were characterized using X-ray diffraction (XRD) analysis performed on a BRUKER D8 ADVANCE diffractometer. The diffraction patterns were registered from 20 to 90 degrees, using Cu Kα radiation (wavelength = 0.15406 nm) at a scanning speed of 2° min^{-1}. The analysis of the crystallographic compositions of the catalysts was performed using the X'Pert HighScore Plus software, and the observed patterns were referenced against the Joint Committee on Powder Diffraction Standards (JCPDS) database for identification.

Transmission electron microscopy (TEM) images were obtained using an FEI TECNAI G2 F20 High-Resolution Transmission Electron Microscope operated at 200 kV. The analysis of these images, specifically for determining the average size of the nanoparticles, was carried out using ImageJ software. Energy-dispersive X-ray spectrometry (EDX) coupled to the TEM was used to determine the chemical compositions, as well as the elemental distribution in the nanoparticles.

XAFS spectra were recorded in transmission mode at the Pd K-edge and in fluorescence mode at the Ni K-edge on beamline B18 at Diamond Light Source (UK). The samples were prepared as pellets by mixing the ground sample into a homogenous mixture with cellulose. Three spectra were averaged for each sample and aligned to their respective metal foils. The data was analysed using Athena and Artemis packages [2] which use the FEFF6 and IFEFFIT codes [3]. Pd fits were carried out in the k range of 3.00 – 12 $Å^{-1}$ and an R range of 1.0 – 3.2 Å. Rh fits were carried out in the 3.00 – 10 $Å^{-1}$ k range and 1.0 – 3.0 Å R range. All fits were done with multiple k weightings 1, 2 and 3. During the fits, the coordination numbers (CN), interatomic distances, Debeye-Waller (DW) factors and energy shifts were refined for each shell while the amplitude reduction factor was fixed at a value from the fitted the metal foil.

Electrochemical characterization

The electrochemical characterizations were carried out at room temperature in a three-electrode glass cell with a single compartment using an Autolab model 302N potentiostat/galvanostat. A hydrogen reference electrode, prepared in the same electrolytic solution and connected via a Luggin capillary, along with a Pt wire counter electrode, was used. The working electrode used was a glassy carbon electrode (0.071 cm^2) coated with the synthesized materials. All potentials were reported relative to the reversible hydrogen electrode (RHE) scale.

The glassy carbon electrode was initially polished using Al_2O_3 (1.0 µm) for cleaning, followed by sonication in 2-propanol for 1 minute to remove residual Al_2O_3. The catalyst ink was prepared by dispersing 3.0 mg of the catalyst in 30 µL of Nafion® and 1000 µL of 2-propanol. This mixture was then sonicated for 10 minutes using a probe-type ultrasonic device (model Eco-sonics) until a homogeneous black ink was formed. Next, 5 µL of this ink was deposited onto the working electrode surface and allowed to air dry at room temperature.

Once dried, the working electrode was inserted into the electrochemical cell, and nitrogen gas (99.99% purity) was bubbled through the electrolyte solution for 15 minutes to ensure a deoxygenated environment. Electrochemical measurements started with 25 cyclec voltammetry sweeps in a 0.5 mol L^{-1} H_2SO_4 solution, ranging from 0.05 to 1.20 V at a scan rate of 200 mV s^{-1}, to stabilize the electrochemical response. Two additional voltammetric profiles were recorded under the same conditions, but in the presence and absence of 0.5 mol L^{-1} formic acid, at a slower scan rate of 20 mV s^{-1} to explore the catalytic behavior.

Chronoamperometric experiments were performed at 0.2 V for 900 seconds. Additionally, CO stripping voltammograms were obtained after saturating the cell with CO gas for 5 minutes at a potential of 0.05 V, followed by purging with N_2 for 15 minutes to remove any dissolved CO. Two voltammograms were then recorded from 0.05 to 1.20 V at 20 mV s^{-1} in a 0.5 mol L^{-1} H_2SO_4 solution. These experiments aimed to evaluate the tolerance of the catalysts to CO and determine the electroactive surface area. Specific activities (mA cm^{-2}) were calculated by dividing the peak current values by the electroactive surface area.

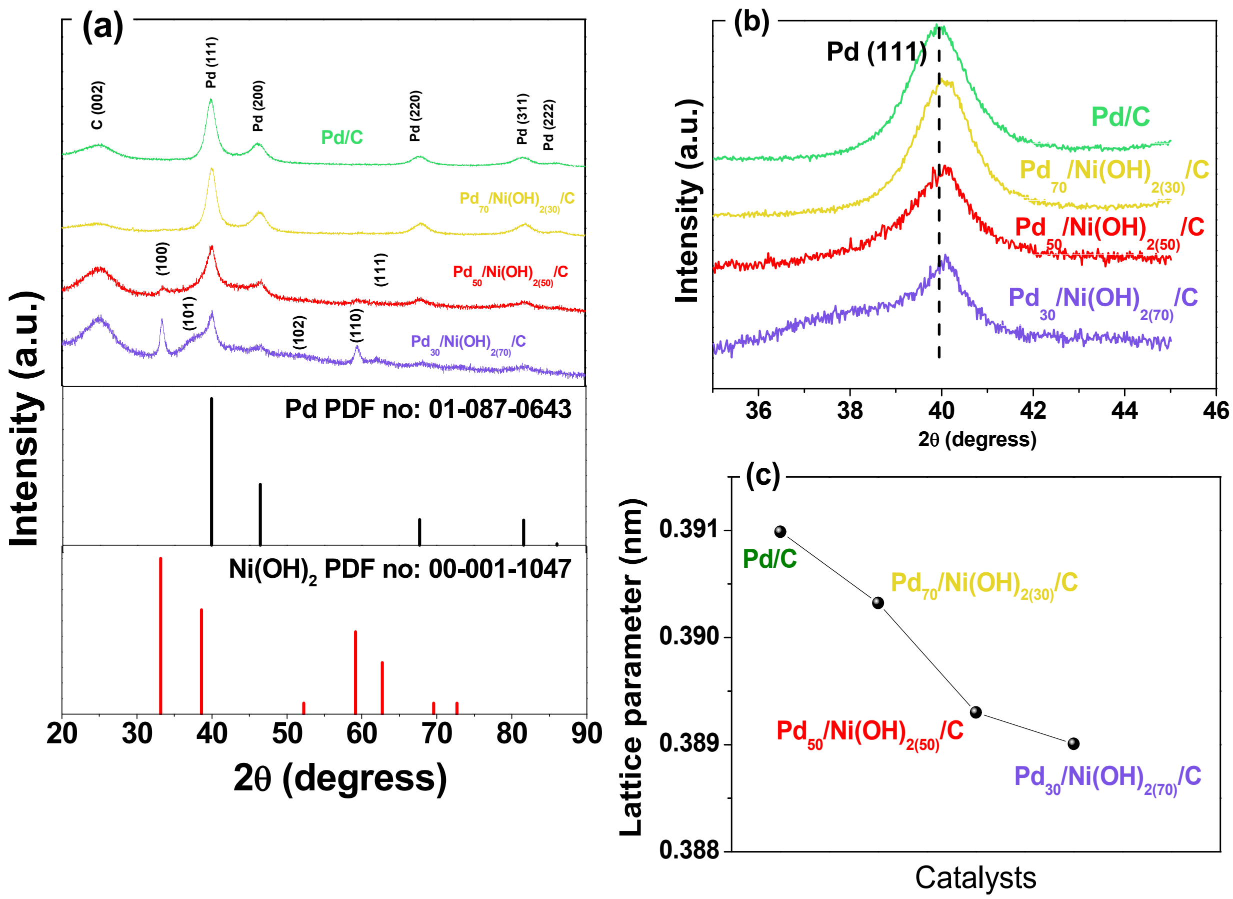

Figure S1. (a) X-ray diffraction patterns of the catalysts Pd/C, $Pd_{70}/Ni(OH)_{2(30)}/C$, $Pd_{50}/Ni(OH)_{2(50)}/C$, Pd_{50}-$Ni(OH)_{2(50)}/C$, and $Pd_{30}/Ni(OH)_{2(70)}/C$. (b) Magnified view of the Pd (111) peak region indicating its displacement for the $Pd_x/Ni(OH)_{2(y)}/C$ materials. (c) Lattice parameters of the different catalysts.

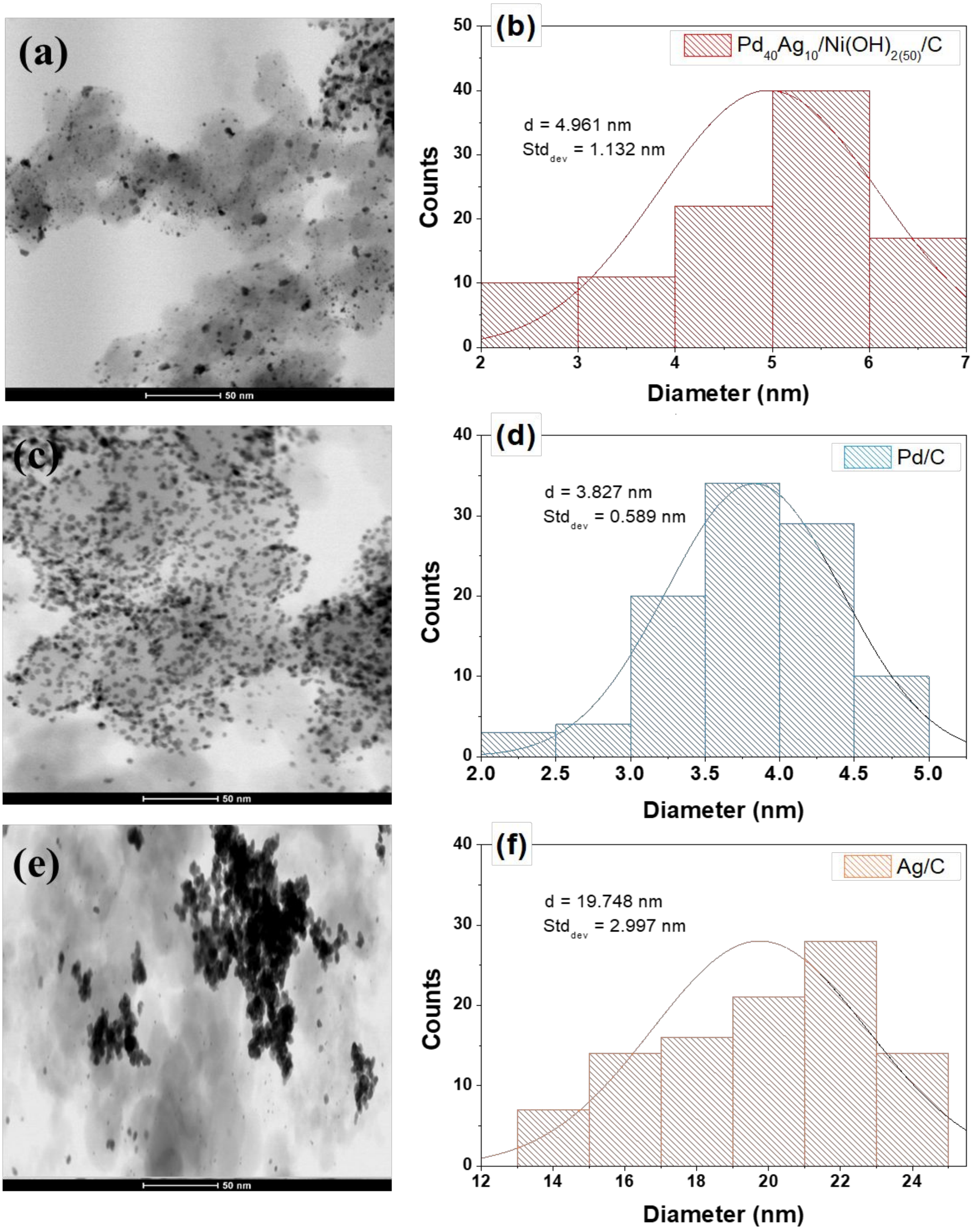

Figure S2. (a, c, e) TEM images and (b, d, f) histograms showing the average particle size distribution (d_m) and standard deviation (Stdev) for $Pd_{40}Ag_{10}Ni(OH)_{2(50)}/C$, Pd/C, and Ag/C, respectively. These histograms were derived from the analysis of 100 nanoparticles to estimate the average particle size.

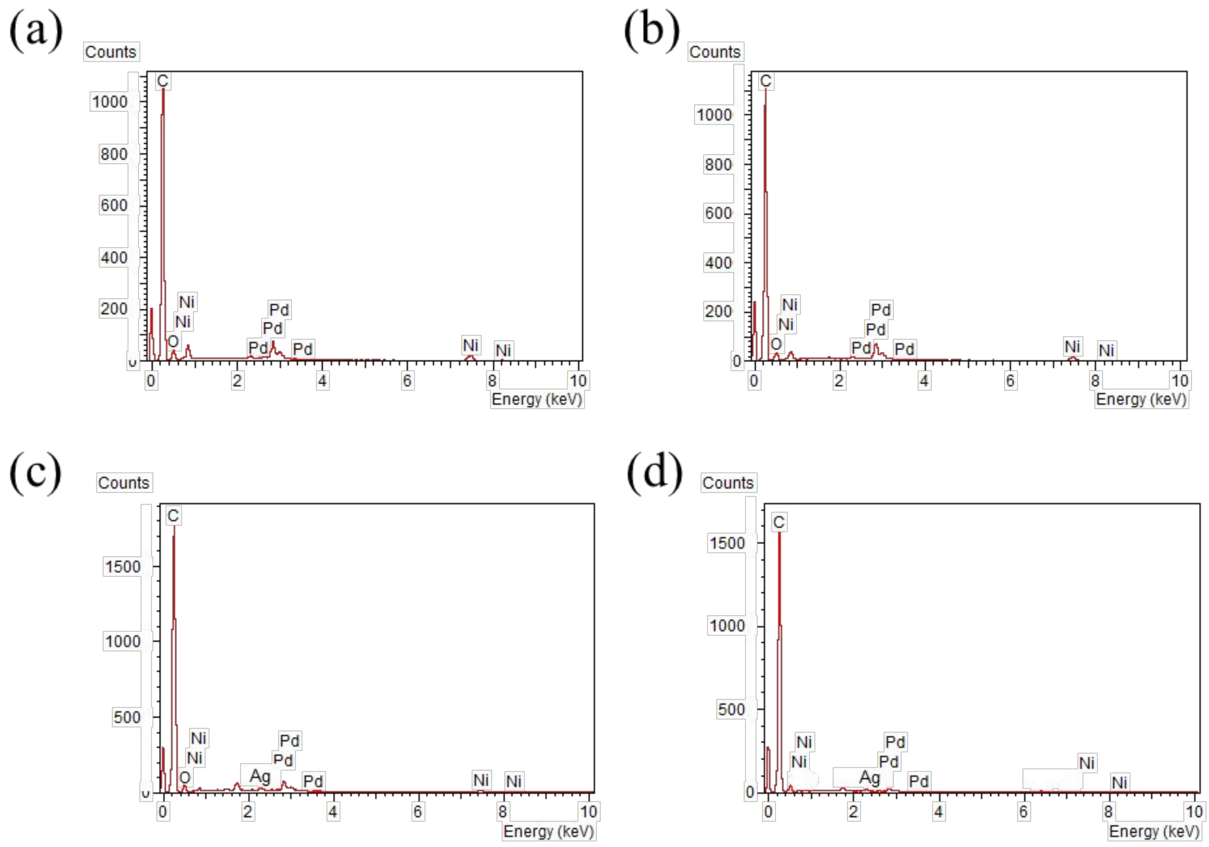

Figure S3. Chemical composition obtained by TEM-EDX analysis for (a) $Pd_{50}/Ni(OH)_{2(50)}/C$, (b) $Pd_{50}Ni(OH)_{2(50)}/C$, (c) $Pd_{30}Ag_{20}/Ni(OH)_{2(50)}/C$, and (d) $Pd_{30}Ag_{20}Ni(OH)_{2(50)}/C$.

Table S1. Summary of chemical composition obtained by TEM-EDX analysis.

**Catalyst**	**Nominal elemental ratio Pd:Ni:Ag**	**Elemental ratio detected by TEM/EDX**		
		Pd	Ni	Ag
**$Pd_{50}/Ni(OH)_{2(50)}/C$**	50:50:0	51.85	48.15	0
**$Pd_{50}Ni(OH)_{2(50)}/C$**	50:50:0	53.14	46.86	0
**$Pd_{30}Ag_{20}/Ni(OH)_{2(50)}/C$**	30:20:50	25.18	56.81	18.09
**$Pd_{30}Ag_{20}Ni(OH)_{2(50)}/C$**	30:20:50	25.56	52.66	21.78

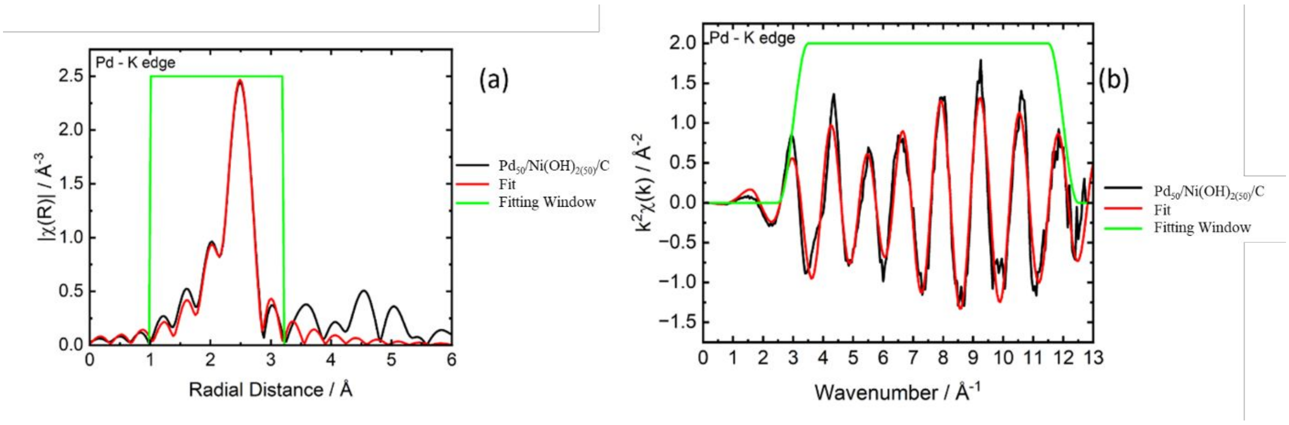

Figure S4 Data and fits of the k^2 weighted Pd K-edge FT signal (a) and k-space plots (b) for the $Pd_{50}/Ni(OH)_{2(50)}/C$ catalyst pellet.

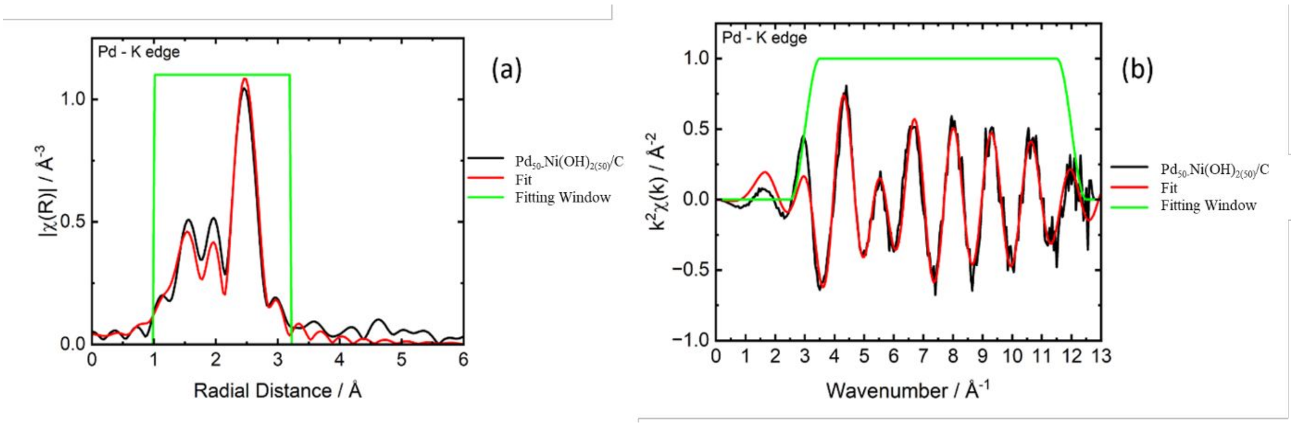

Figure S5 Data and fits of the k^2 weighted Pd K-edge FT signal (a) and k-space plots (b) for the Pd_{50}-$Ni(OH)_{2(50)}/C$ catalyst pellet.

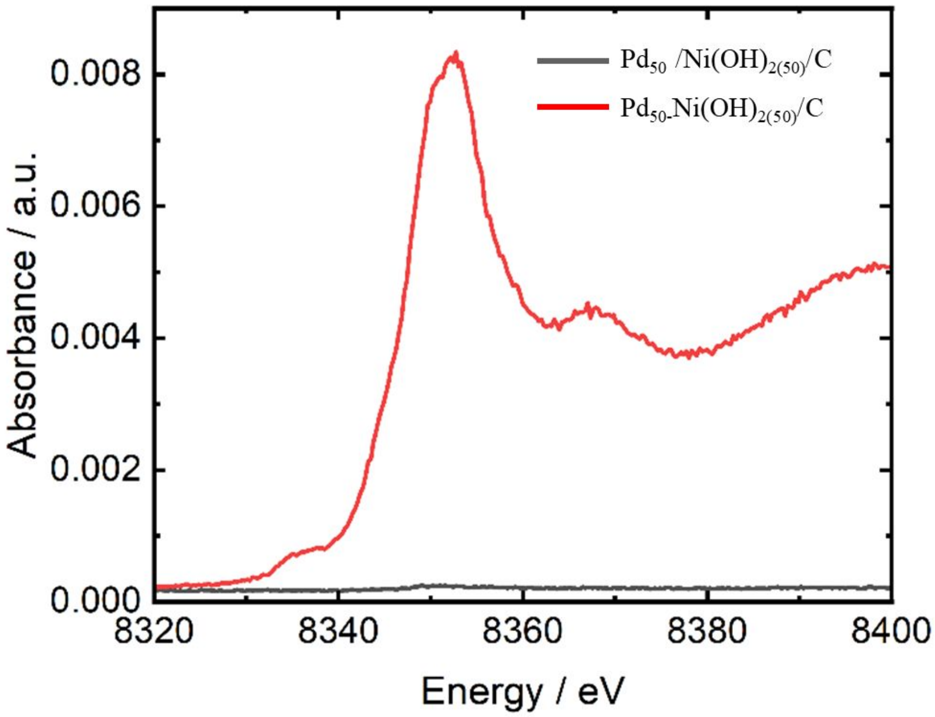

Figure S6 Ni K edge XANES of the absolute absorbance of $Pd_{50}/Ni(OH)_{2(50)}/C$ and Pd_{50}-$Ni(OH)_{2(50)}/C$

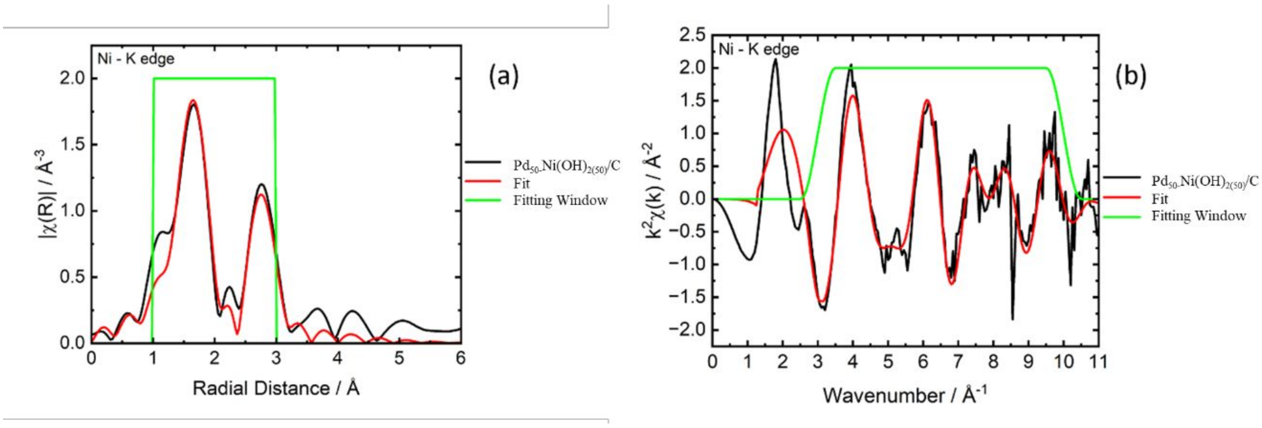

Figure S7 Data and fits of the k^2 weighted Ni K-edge FT signal (a) and k-space plots (b) for the Pd_{50}-$Ni(OH)_{2(50)}/C$ catalyst pellet.

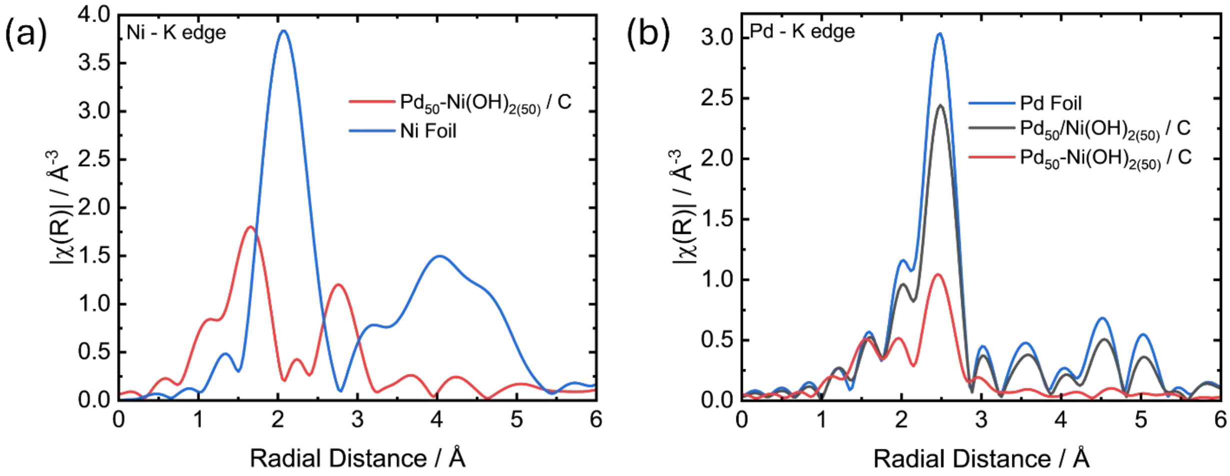

Figure S8 Data and comparison with reference spectra of Ni and Pd foil of the k^2 weighted Ni K-edge FT signal for the Pd_{50}-$Ni(OH)_{2(50)}$/C catalyst pellet (a) and Pd K-edge FT signal for the Pd_{50}/$Ni(OH)_{2(50)}$/C and Pd_{50}-$Ni(OH)_{2(50)}$/C catalyst pellet.

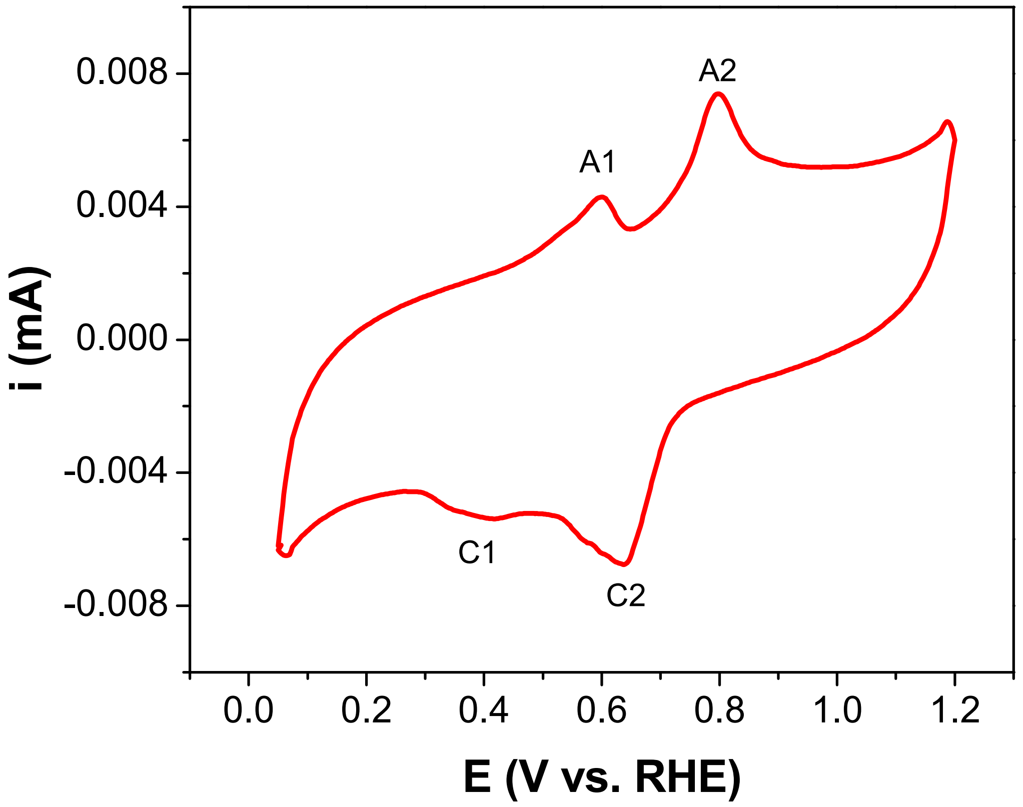

Figure S9. (a) Cyclic voltammograms (second cycle) for in 0.5 mol L^{-1} in H_2SO_4 at 25 °C for Ag/C at υ = 20 mV s^{-1}. The anodic and cathodic peaks are associated with the formation of Ag_2O (A1) and AgO (A2) and their reduction (C1 and C2).

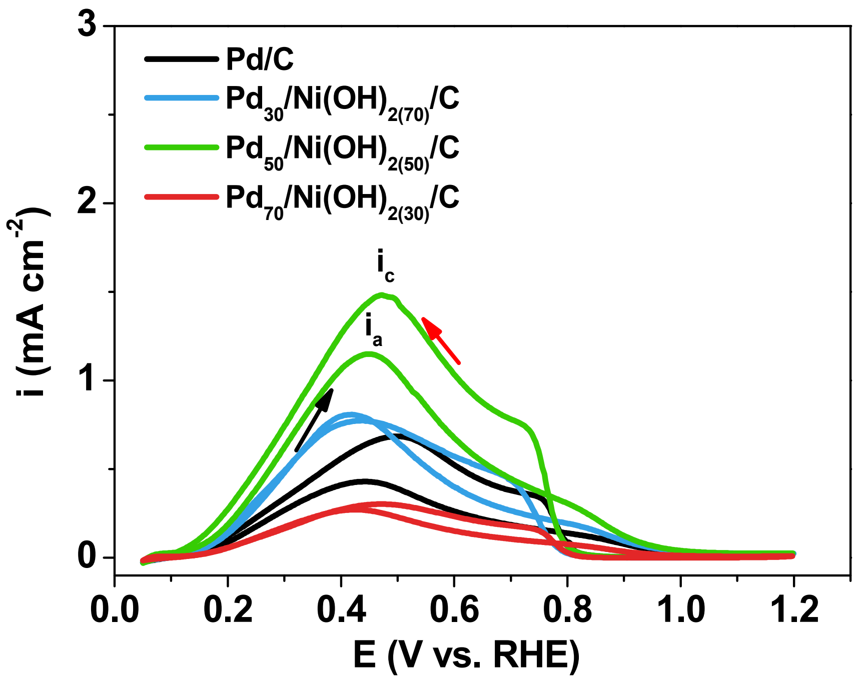

Figure S10. (a) Cyclic voltammograms (second cycle) for formic acid oxidation (0.5 mol L^{-1} in H_2SO_4) at 25 °C for the catalysts Pd/C and $Pd_x/Ni(OH)_{2(1-x)}/C$. υ = 20 mV s^{-1}. Peaks for formic acid oxidation are denoted as i_a (anodic) and i_c (cathodic), with arrows indicating scan direction: () anodic and () cathodic.

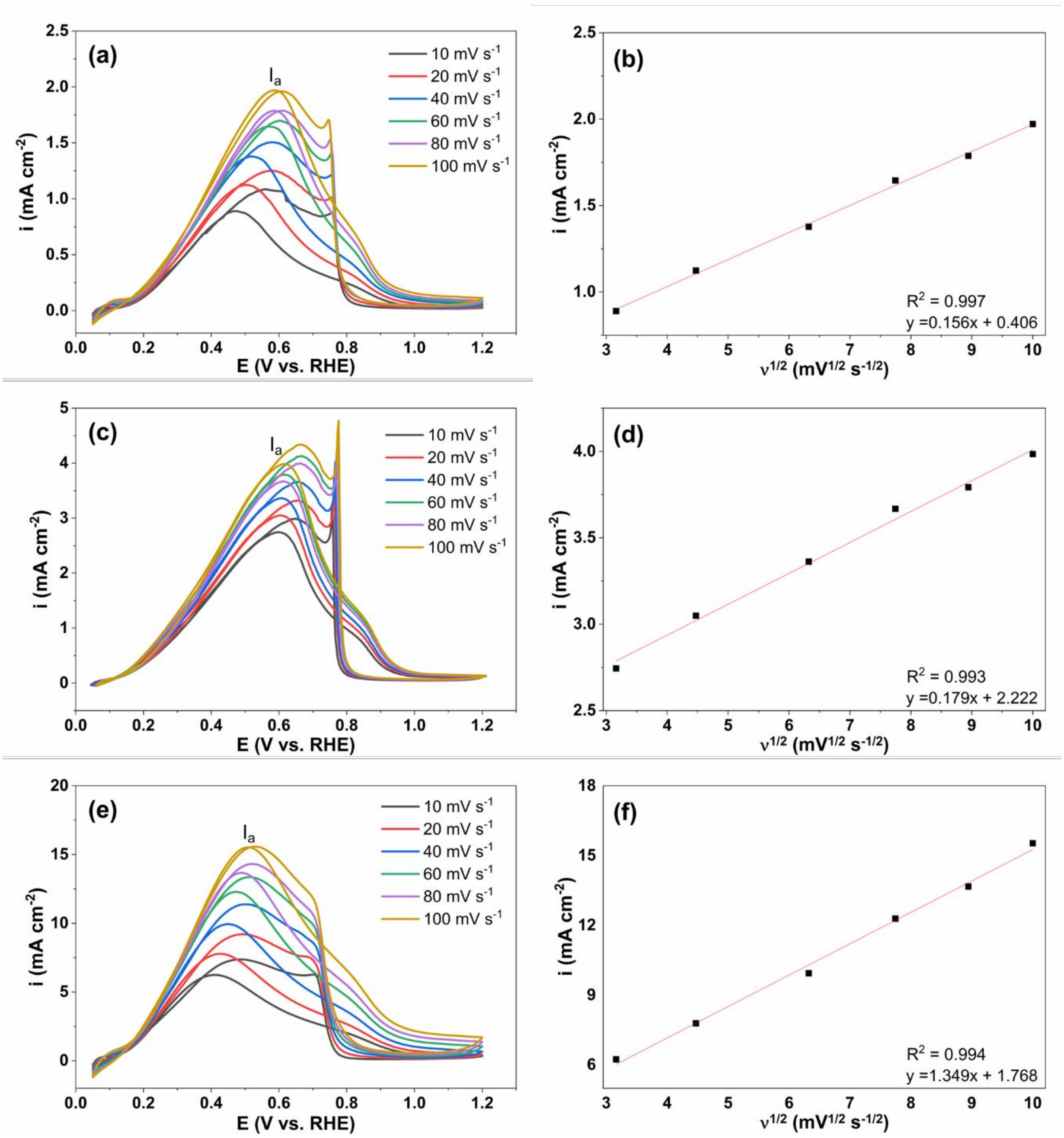

Figure S11. Cyclic voltammograms (2nd cycle) at different scan rates (10, 20, 40, 60, 80 and 100 mV s^{-1}) and the relationship between the anodic scan current density (I_a) and the square root of the scan rate ($\nu^{1/2}$) for (a, b) Pd/C, (c, d) $Pd_{50}Ni(OH)_{2(50)}$/C and (e, f) $Pd_{30}Ag_{20}Ni(OH)_{2(50)}$/C in 0.5 mol L^{-1} H_2SO_4 and 0.5 mol L^{-1} formic acid solutions.

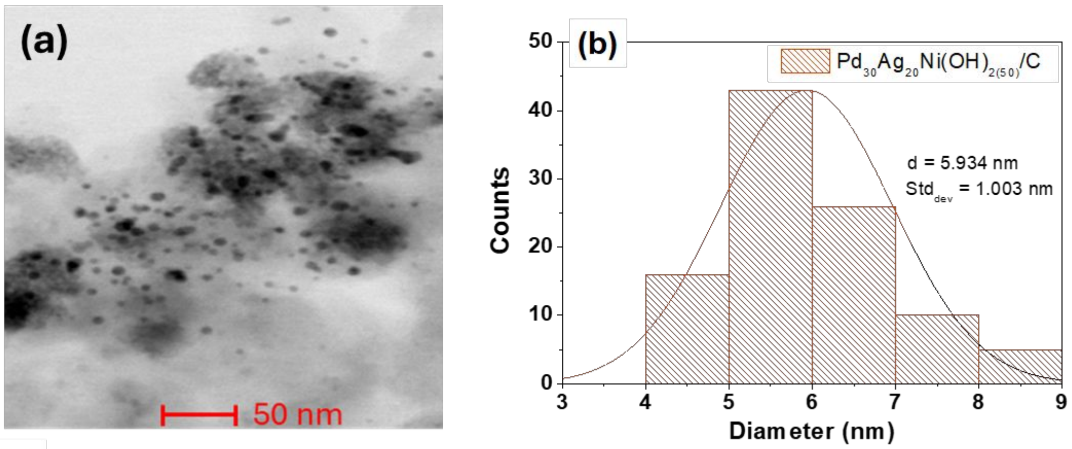

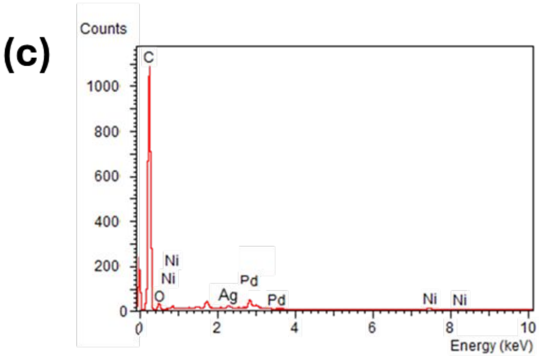

Before stability test			After stability test		
Elemental ratio detected by TEM/EDX			Elemental ratio detected by TEM/EDX		
Pd	Ni	Ag	Pd	Ni	Ag
25.56	52.66	21.78	34.42	49.73	15.85

Figure S12. (a) TEM image and histogram (b) showing the average particle size distribution (dm) and standard deviation (Stdev) for $Pd_{30}Ag_{20}Ni(OH)_{2(50)}/C$ after the stability test. These histograms were derived from the analysis of 100 nanoparticles to estimate the average particle size. (c) Chemical composition obtained by TEM-EDX analysis and the comparison of chemical composition before and after stability test.

References

[1] C.V.S. Almeida, K.I.B. Eguiluz, G.R. Salazar-Banda, Superior ethanol electrooxidation activity of Pd supported on $Ni(OH)_2/C$. The effect of $Ni(OH)_2$ nanosheets content, J. Electroanal. Chem. 878 (2020) 114683.

[2] B. Ravel, M. Newville, ATHENA, ARTEMIS, HEPHAESTUS: data analysis for X-ray absorption spectroscopy using IFEFFIT, J. Synchrotron Radiat. 12 (2005) 537-541

[3] M. Newville, EXAFS analysis using FEFF and FEFFIT, J. Synchrotron Radiat. 8 (2001), 96-100.
